# Interactions between the AraC/XylS-like transcriptional activator InvF of *Salmonella* Typhimurium, the RNA polymerase alpha subunit and the chaperone SicA

**DOI:** 10.1038/s41598-023-50636-w

**Published:** 2024-01-02

**Authors:** Daniel Cortés-Avalos, André Borges Farias, Luis E. Romero-González, Cristina Lara-Ochoa, Lourdes Villa-Tanaca, Francisco García-del Portillo, Vanessa López-Guerrero, Víctor H. Bustamante, Ernesto Pérez-Rueda, J. Antonio Ibarra

**Affiliations:** 1 Laboratorio de Genética Microbiana, Departamento de Microbiología, Escuela Nacional de Ciencias Biológicas, Instituto Politécnico Nacional. Prol. Carpio y Plan de Ayala S/N, Col. Santo Tomás 11340, Mexico City, Mexico; 2 Instituto de Investigaciones en Matemáticas Aplicadas y en Sistemas, Universidad Nacional Autónoma de México, Unidad Académica del Estado de Yucatán, Mérida, Mexico; 3 Departamento de Microbiología Molecular, Instituto de Biotecnología, Universidad Nacional Autónoma de México, Cuernavaca, Morelos Mexico; 4 Centro de Detección Biomolecular, Benemérita Universidad Autónoma de Puebla, Puebla, Mexico; 5 Laboratory of Intracellular Bacterial Pathogens, National Centre for Biotechnology (CNB)-CSIC, Darwin, 3, 28049 Madrid, Spain; 6 Facultad de Medicina, Universidad Autónoma del Estado de Morelos, Cuernavaca, Morelos Mexico

**Keywords:** DNA-binding proteins, Transcriptional regulatory elements, Chaperones

## Abstract

*Salmonella enterica* serovar Typhimurium causes gastroenteritis and systemic infections in humans. For this bacterium the expression of a type III secretion system (T3SS) and effector proteins encoded in the *Salmonella* pathogenicity island-1 (SPI-1), is keystone for the virulence of this bacterium. Expression of these is controlled by a regulatory cascade starting with the transcriptional regulators HilD, HilC and RtsA that induce the expression of HilA, which then activates expression of the regulator InvF, a transcriptional regulator of the AraC/XylS family. InvF needs to interact with the chaperone SicA to activate transcription of SPI-1 genes including *sicA, sopB, sptP, sopE, sopE2*, and *STM1239*. InvF very likely acts as a classical activator; however, whether InvF interacts with the RNA polymerase alpha subunit RpoA has not been determined. Results from this study confirm the interaction between InvF with SicA and reveal that both proteins interact with the RNAP alpha subunit. Thus, our study further supports that the InvF/SicA complex acts as a classical activator. Additionally, we showed for the first time an interaction between a chaperone of T3SS effectors (SicA) and the RNAP.

## Introduction

**Figure 1:**
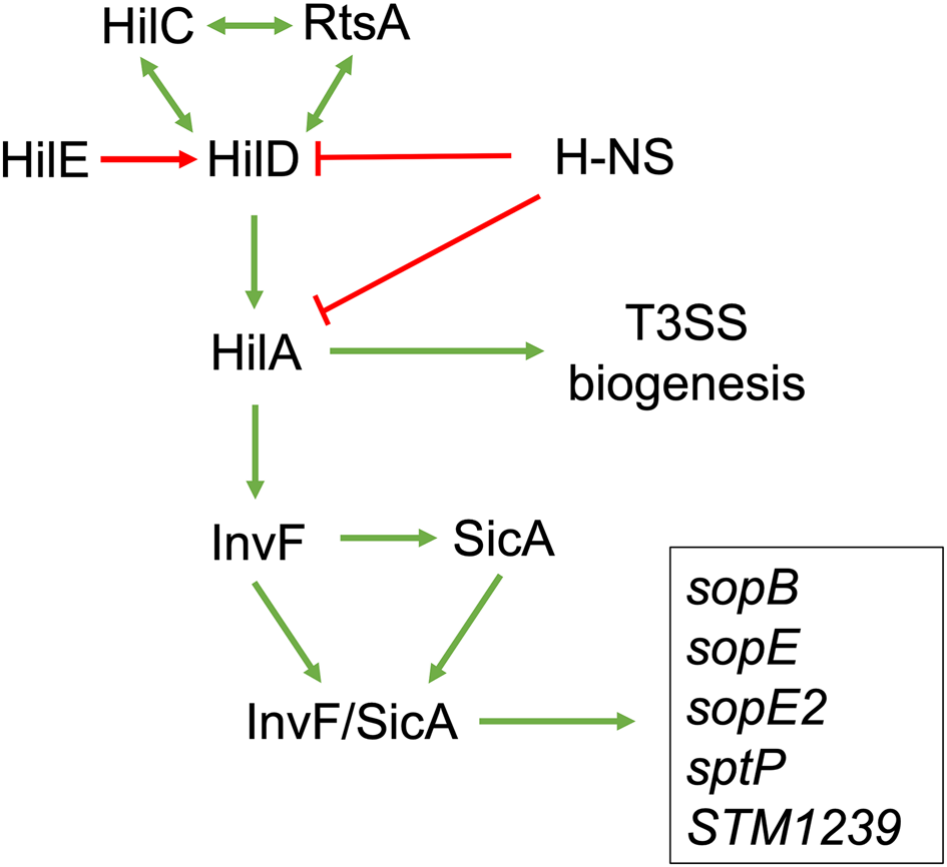
Simplified representation of the SPI-1 regulatory cascade. The expression of SPI-1 is controlled by a regulatory network where the transcriptional regulators HilD, HilC and RtsA form a feed-forward loop that activates HilA. HilE interacts with HilD inactivating this regulator while H-NS downregulates HilD and HilA. HilA then activates the expression of genes involved in the biosynthesis of the T3SS-1 and, also activates InvF, the last transcriptional activator of this cascade. InvF both regulates expression of the chaperone SicA and interacts with it. The InvF/SicA complex regulates expression of many effector genes included *sopB, sptP, sopE, sopE2, STM1239*. Green arrows represent gene expression activation, red blunted lines represent repression of expression and red arrows represent inactivation.

The genus *Salmonella* contains pathogenic bacteria that infect humans and animals; for instance, *Salmonella enterica* serovar Typhimurium (STM) can cause gastroenteritis and systemic infections in both humans and other mammals^1,2^. STM has many virulence genes clustered in regions called *Salmonella* pathogenicity islands^3^. Both *Salmonella* pathogenicity islands 1 and 2 (SPI-1 and SPI-2) encode a type III secretion system (T3SS-1 and T3SS-2, respectively), effector proteins, chaperones and transcriptional regulators that control genes within and outside these islands^4,5^. The T3SS-1 is required for invasion and replication of *Salmonella* in the cytosol of epithelial cells while the T3SS-2 is necessary for survival and replication within the *Salmonella* containing vacuole (SCV)^6^. The expression of the T3SS-1 is controlled by a complex regulatory network where the transcriptional regulators HilD, HilC and RtsA form a positive feed-forward loop that activates *hilA* transcription, HilA in turn activates the expression of several genes involved in the biosynthesis of the T3SS-1 and also that of InvF, a transcriptional regulator that belongs to the AraC/XylS family^7^. InvF interacts with SicA, a chaperone protein that binds to multiple effector proteins and T3SS-1 components^8–10^. Finally, the InvF/SicA complex activates transcription of many genes including *sicA, sopB, sptP, sopE, sopE2, STM1239* and genes encoding for components of the T3SS-1 (Fig. 1)^11,12^. Thus, InvF represents an important transcriptional regulator for STM pathogenesis.

Previous findings support that InvF acts as classical transcriptional regulator^9,10^. However, it is not clear whether InvF interacts with the RNA polymerase (RNAP) to activate gene transcription as other classical regulators from the AraC/XylS family, such as PerA, MelR and XylS^13–15^. In this report we demonstrate that both InvF and SicA interact with the RNAP machinery through the alpha subunit in vitro and in vivo, further deciphering the mechanism by which the InvF/SicA complex induces gene expression.

## Results

### InvF interacts with the RNA polymerase alpha subunit

**Figure 2:**
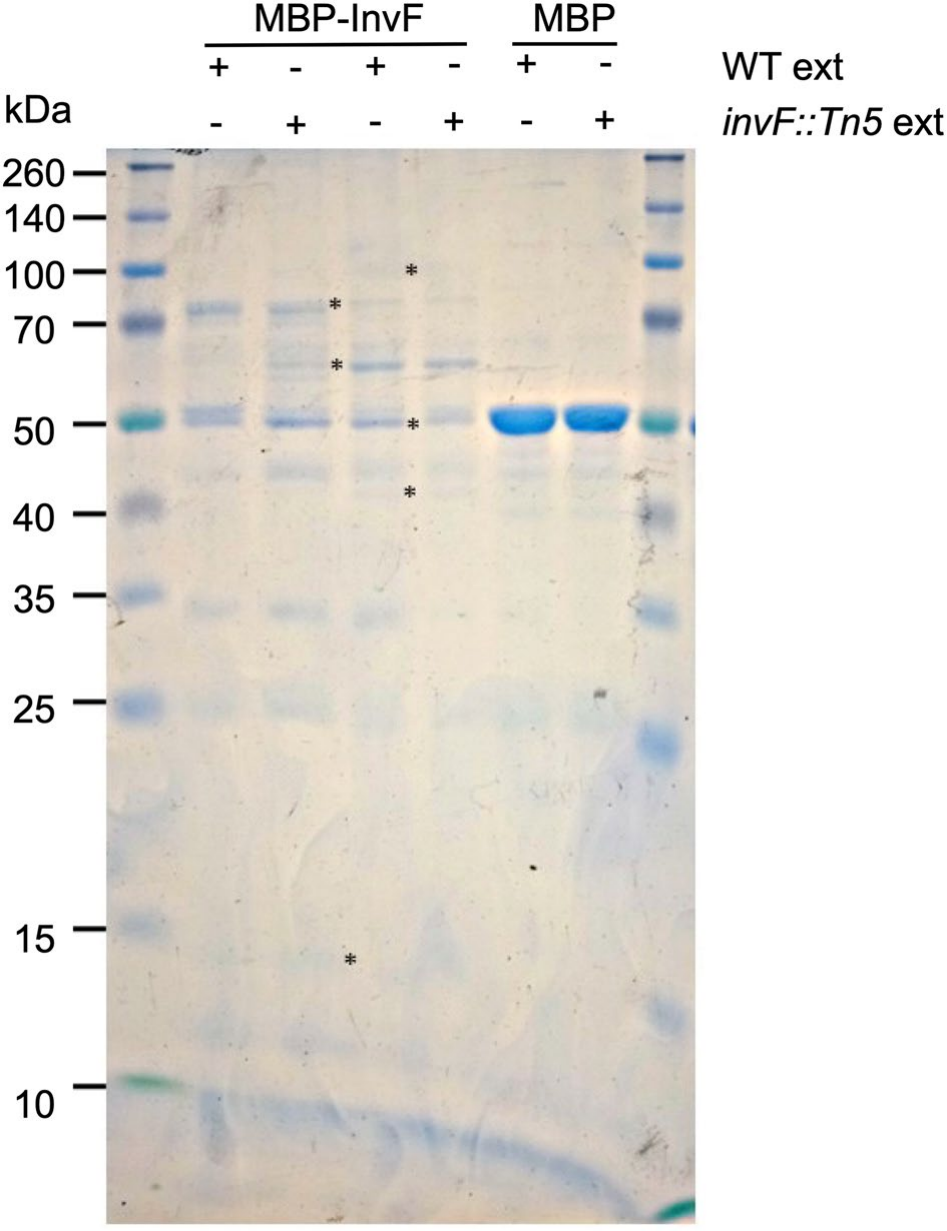
MBP-InvF protein–protein interactions with *Salmonella* extracts. Pulled down proteins were separated by SDS-PAGE and selected bands were excised (shown with asterisks). Molecular weight markers are shown in each side of the gel, while the use or not of either MBP-InvF or MBP is shown with ( +) and (−), respectively. Either the wild type STM (WT ext) or the STM *invF* mutant (*invF::Tn5* ext) extracts used in each interaction is indicated.

To detect probable protein–protein interactions of InvF with other cytoplasmic proteins, pull-down assays were performed with a recombinant version of InvF fused to the maltose-binding protein (MBP-InvF) as the bait and cell-free soluble extracts obtained from STM wild type and its derivative *invF::Tn5* mutant grown in SPI-1-inducing conditions^16^. Purified MBP was assessed as a bait negative control. Interacting pulled down proteins were analyzed by SDS-PAGE, proteins captured with MBP-InvF, but not with MBP were selected and analyzed by LC/MS–MS (Fig. 2 and Supplementary File 1). Among the detected proteins interacting with MBP-InvF multiple subunits of the RNAP were identified, such as the beta, sigma and alpha subunits, suggesting interactions with at least one of them (Table 1). These results suggest that InvF interacts with the RNAP.

**Table 1:** Summary of identified proteins interacting with InvF and SicA.

Molecular weight (kDa)^a^	Identified proteins^b^	Accession number (GenBank)	Counts^c^
100	DNA-directed RNA polymerase subunit beta	WP_000263106.1	36
	DNA-directed RNA polymerase subunit beta’	CBW20181	49
	RNA polymerase sigma factor RpoD	CBW19283.1	26
	Protein disaggregation chaperon ClpB	WP_001235094.1	239
	ATP-dependent Clp protease ATP-binding subunit ClpA	WP_000934063.1	71
70	Transcriptional regulator InvF	WP_001674874.1	256
	Maltose binding protein	CBW20253.1	127
40	Transcriptional regulator InvF	WP_001674874.1	10
	Maltose binding protein	CBW20253.1	135
	DNA-directed RNA polymerase subunit alpha	WP_001162094.1	35
	Carboxipeptidase	CBW16725.1	19

^a^Molecular size as determined by SDS-PAGE.

^b^Protein was identified by LC–MS/MS.

^c^Number of times peptides from these proteins were identified.

**Figure 3:**
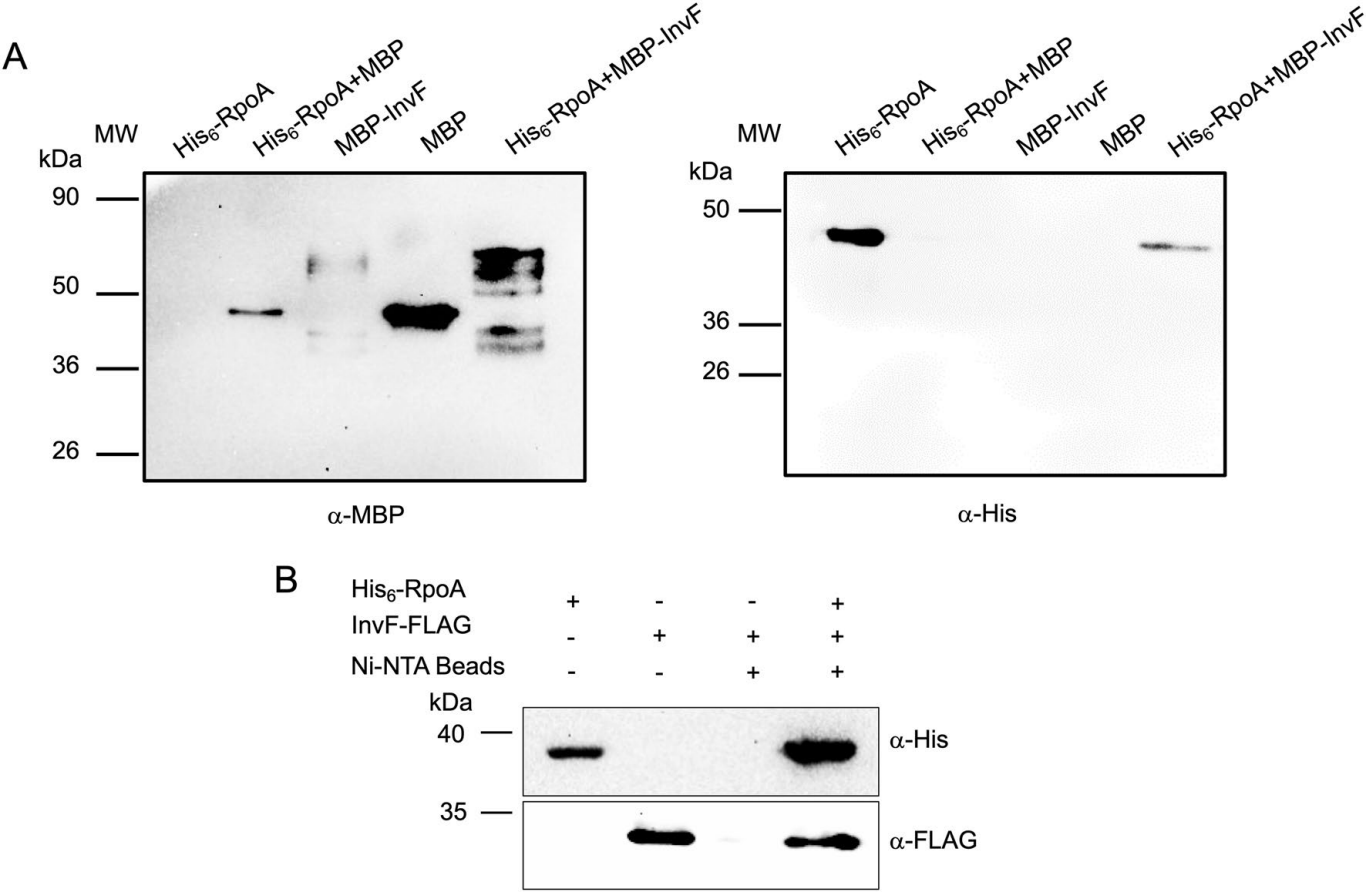
The regulator InvF interacts with RpoA. (**A**) Pull-down assay to detect in vitro InvF-RpoA interactions. A pull-down assay using MBP or MBP-InvF proteins and purified His_6_-RpoA was performed. Left panel shows the control proteins (His_6_-RpoA, MBP and MBP-InvF) and the interaction reactions His_6_-RpoA + MBP and His_6_-RpoA + MBP-InvF detected with anti-MBP antibodies. Amylose resin was used for the pull-down reactions. Right panel shows the detection of His_6_-RpoA with a His-probe (α-His). (**B**) Pull-down to detect in vivo the InvF-RpoA interaction. Cell-free extracts of *Salmonella* expressing InvF-FLAG and His_6_-RpoA from plasmid pET28-RpoA was pulled down with Ni–NTA resin. The purified protein His_6_-RpoA and the cell-free extract were used as controls. Proteins were detected by Western blot with His-Probe and anti-FLAG-HRP. Cell-free extracts were obtained from the indicated cultures in SPI-1-inducing conditions as described in the “Methods” section. Experiments were performed in triplicate.

Previous studies have shown that other members of the AraC/XylS family of transcriptional regulators interact with the alpha subunit of the RNAP (RpoA)^13,14,17–26^. To determine whether InvF binds to RpoA multiple approaches were followed. Initially, pull-down experiments between InvF and RpoA were performed using purified MBP-InvF or His_6_-RpoA proteins and with cell-free soluble extracts of the STM *invF*::*3xFLAG* strain transformed with the pET28-RpoA plasmid. Results showed that in vitro MBP-InvF was able to interact with His_6_-RpoA (Fig. 3A) and a similar result was observed in vivo (Fig. 3B) when InvF-FLAG and His_6_-RpoA (bait) were used. These results demonstrated that the regulator MBP-InvF interacts with His_6_-RpoA.

**Figure 4:**
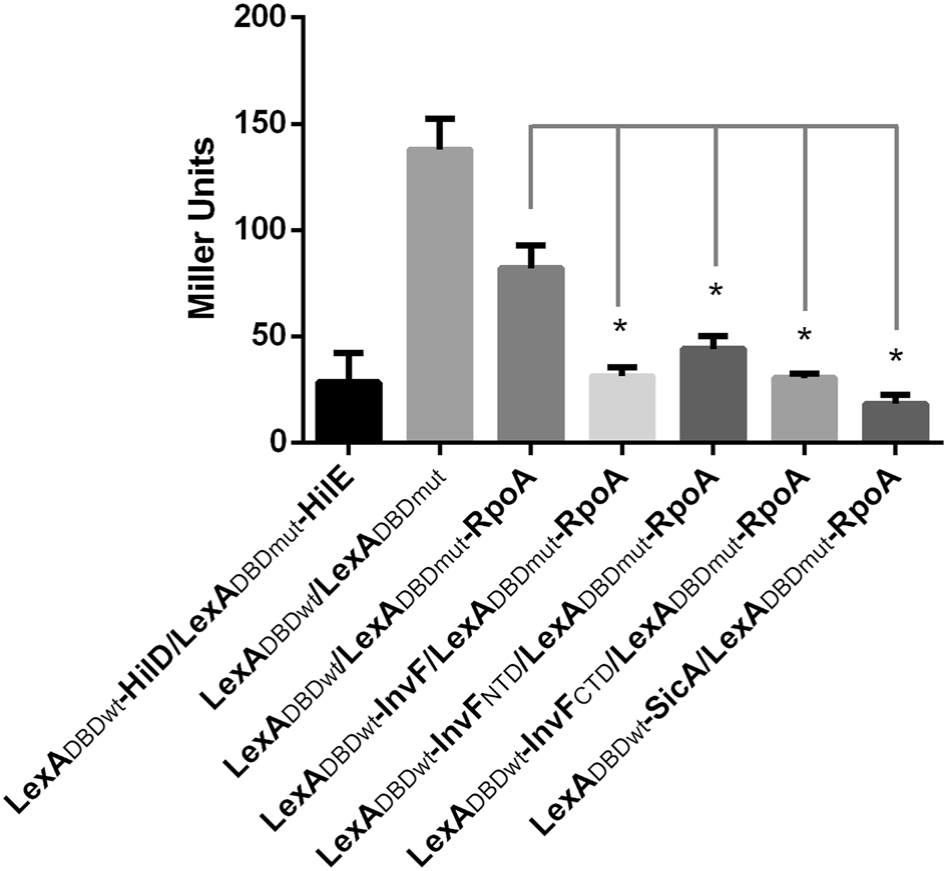
RpoA interactions with InvF and SicA detected with a LexA-based two hybrid system. β-galactosidase activity of *E. coli* SU202 strains transformed with LexA-derivative plasmids grown and processed as described in the “Methods” section. Constructs are indicated below each bar. Bars represent the average of three independent experiments and the error bars represent the standard deviation. * indicates statistically significant difference (*P* < 0.01) compared to the controls indicated with lines.

Additionally, the interaction between InvF and RpoA was tested by using a bacterial LexA-based two hybrid system^27,28^, which has been successfully used in our laboratories to assess protein–protein interactions^10,29,30^. Briefly, in this bacterial two hybrid system the protein of interest is fused to the wild type LexA DNA binding domain (LexA_DBDwt_) and the construct is transformed into an *E. coli* reporter strain (SU101) encoding a transcriptional fusion *sulA-lacZ*, which has a LexA wild type operator. This system also allows the detection of heterodimers, this is achieved by fusing the other protein of interest to a mutated version of the LexA DNA binding domain (LexA_DBDmut_), then both constructs are transformed into an *E. coli* strain (SU202) encoding a mutated version of the *sulA-lacZ* fusion. In both cases, the expression of LacZ means that there is no protein–protein interaction and, on the contrary, a reduction of the β-galactosidase activity would mean that, and interaction of the fused protein (s) has occurred (homodimerization for SU101 and heterodimerization for SU202)^27,28^. InvF and RpoA were fused to either the wild type or to the mutated LexA DNA binding domain (LexA_DBDwt_ and LexA_DBDmut_, respectively) and the β-galactosidase activity was tested in the reporter strain. Negative controls included the empty vectors and a combination of LexA_DBDmut_-RpoA and LexA_DBDwt_, while the positive control was the chimeric proteins LexA_DBDwt_-HilD and LexA_DBDmut_-HilE. Results in Fig. 4 show that control fusions LexA_DBDwt_-HilD and LexA_DBDmut_-HilE repress the expression of the *sulA-lacZ*, as expected^30^. When the fusion protein LexA_DBDmut_-RpoA was tested with either LexA_DBDwt_-InvF or LexA_DBDwt_-SicA, the expression of the reporter fusion was reduced, indicating that RpoA interacts with both proteins.

**Figure 5:**
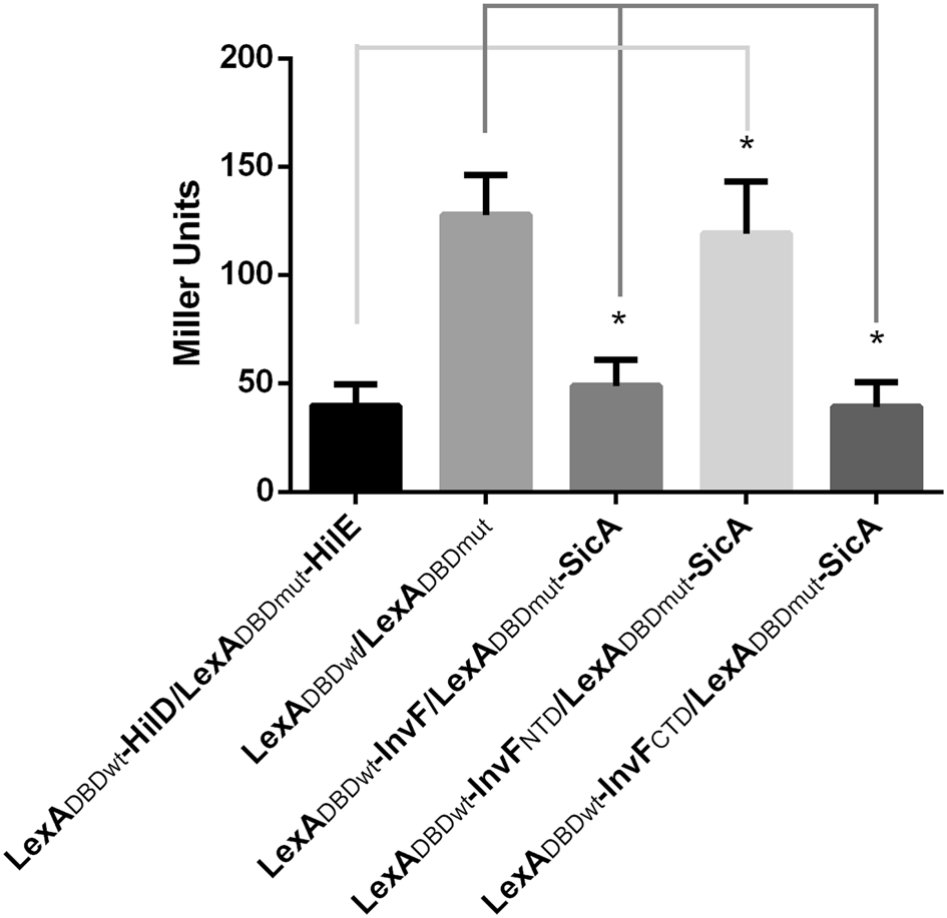
Interactions of InvF and SicA detected with a LexA-based two hybrid system. β-galactosidase activity of *E. coli* SU202 strains transformed with LexA-derivative plasmids grown and processed as described in the “Methods” section. Constructs are indicated below each bar. Bars represent the average of three independent experiments and the error bars represent the standard deviation. * indicates statistically significant difference (*P* < 0.01) compared to the controls indicated with lines.

Subsequently, to determine the region of InvF contacting RpoA, the N-terminal and C-terminal regions of InvF were fused to LexA_DBDwt_ and tested for dimerization with RpoA. Both, the N-terminal (LexA_DBDwt_-InvF_NTD_) and the C-terminal domains (LexA_DBDwt_-InvF_CTD_) domains of InvF interacted with RpoA (LexA_DBDmut_-RpoA) (Fig. 4). The interaction of both InvF domains with SicA was also tested and results showed that InvF C-terminal domain (LexA_DBDwt_-InvF_CTD_) interacts with SicA, while the N-terminal domain (LexA_DBDwt_-InvF_NTD_) does not (Fig. 5). These results confirm that RpoA makes contacts with the whole protein and also independently with both InvF domains.

### SicA interacts with RpoA

**Figure 6:**
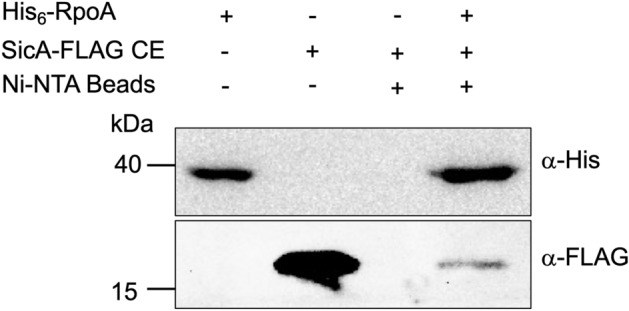
The chaperone SicA interacts with RpoA. Pull-down assays performed with Ni–NTA magnetic beads to detect in vitro SicA-FLAG and His_6_-RpoA interactions. Purified His_6_-RpoA and cell-free extract containing SicA-FLAG were used as controls. The chimeric proteins were detected by Western blot with His-Probe and anti-FLAG-HRP antibodies. Cell-free extracts were obtained from the indicated cultures as described in the “Methods” section. Experiments were performed by triplicate.

Previous evidence indicates that SicA is strictly necessary for InvF to activate transcription of target genes and that both proteins interact^9,10,12,31^. Based on in silico analyses, we recently predicted that SicA makes contacts not only with InvF, but also with RpoA^32^. To investigate this prediction, the interaction between SicA and RpoA was tested by using the LexA-based dimerization system and pull-down experiments. Both approaches showed that SicA interacts with RpoA; the combination LexA_DBDwt_-SicA and LexA_DBDmut_-RpoA repressed the expression of the *sulA-lacZ* fusion in the LexA-based system (Fig. 4), whereas the His_6_-RpoA captured SicA-FLAG in the pull-down assays (Fig. 6). These results show that SicA interacts also with RpoA independently of InvF and supports a model indicating that the three proteins might form a trimeric complex.

### InvF, SicA and RpoA interactions

**Figure 7:**
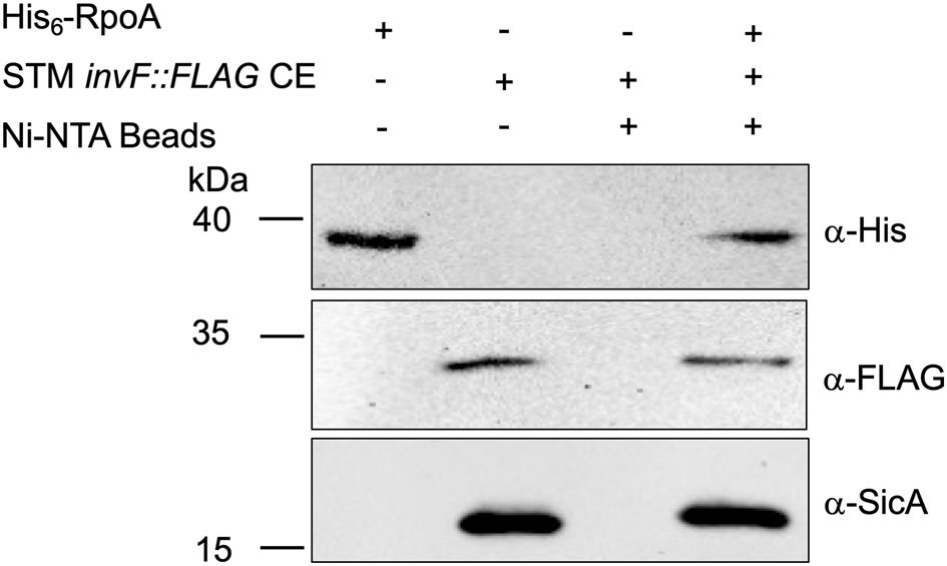
InvF, SicA and RpoA interactions. Pull-down assays performed with Ni–NTA magnetic beads to detect in vivo InvF-RpoA-SicA interactions. Purified His_6_-RpoA and cell-free extract containing InvF-FLAG and SicA were used as controls. Proteins were detected by Western blot with His-Probe, anti-FLAG-HRP and anti-SicA antibodies. Cell-free extracts were obtained from the indicated cultures in SPI-1-inducing conditions as described in the “Methods” section. Experiments were performed by triplicate.

To corroborate whether the three proteins interact forming a ternary complex pull-down experiments were done with cell-free extract of STM *invF::3xFLAG* pET28-RpoA using His_6_-RpoA as bait. Pulled-down proteins were detected by Western blot with either anti-SicA or anti-FLAG-HRP antibodies. Results in Fig. 7 show that both proteins InvF-FLAG and SicA from the extract were detected interacting with His_6_-RpoA suggesting that these three proteins might be forming a complex in solution.

### RpoA α-CTD is important for *sopB* expression

**Figure 8:**
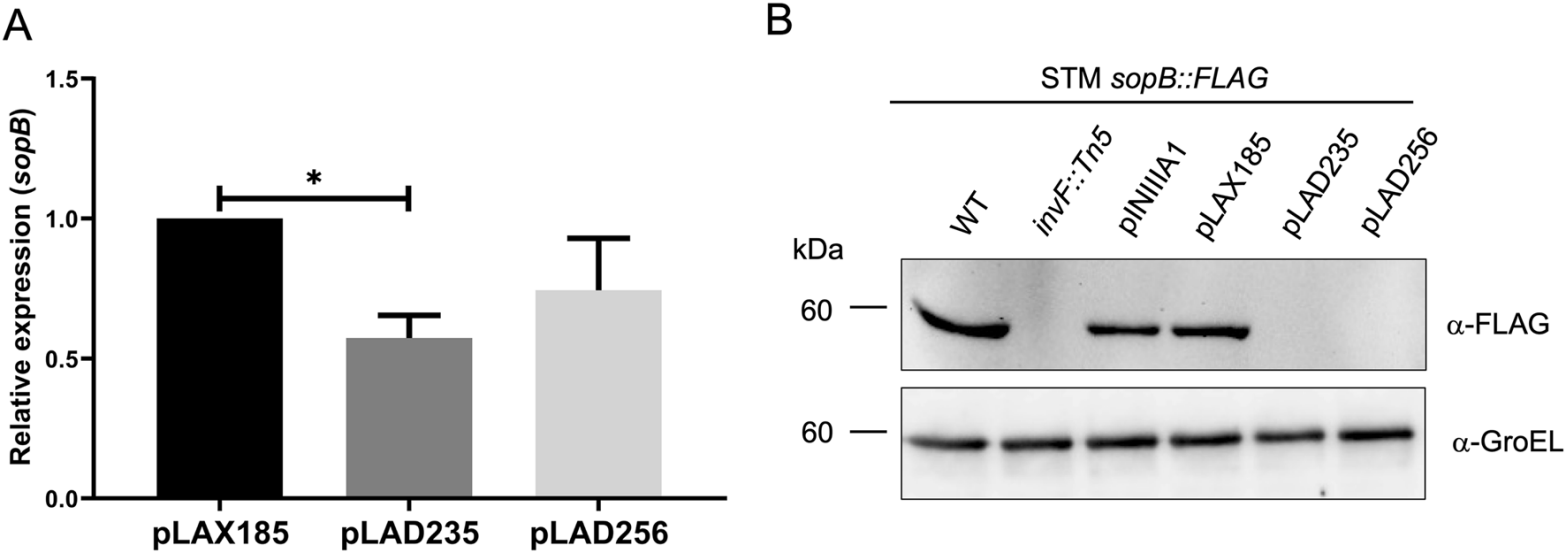
The *sopB* expression is affected by RpoA negative dominant mutants. (**A**) The differential expression of the InvF-dependent gene, *sopB*, was analyzed by RT-qPCR in *Salmonella* Typhimurium transformed with plasmids pLAX185 (wild type *rpoA*), pLAD235 (*rpoA*Δ235 mutant) and pLAD256 (*rpoA*Δ256 mutant) grown in SPI-1-inducing conditions. Data represent the means of three different experiments. The bars indicate the standard deviation. Expression analysis was performed using the ^ΔΔ^Ct method. *. P < 0.05. (**B**) SopB expression in *Salmonella* Typhimurium (SMT) *sopB::FLAG* strains transformed with pNIIIA1, pLAX185, pLAD235 and pLAD256 grown in SPI-1-inducing conditions. GroEL was used as load control. SopB-FLAG and GroEL were detected by Western blot with anti-FLAG-HRP and anti-GroEL antibodies. The figure shows one of the three replicates done.

To investigate whether the RpoA carboxy-terminal domain (α-CTD) is involved in gene expression mediated by InvF/SicA, we analyzed the effect of two negative dominant versions of RpoA on the expression of *sopB*. Plasmids pLAD235 and pLAD256, expressing RpoA negative dominant mutants lacking different portions of α-CTD, were transformed in wild type STM and the expression of *sopB* was tested by RT-qPCR in samples of bacterial cultures grown in SPI-1-inducing conditions^33^. Expression of *sopB* decreased with both mutants (Fig. 8A). Additionally, the expression of SopB-FLAG in the presence of the RpoA negative dominant mutants was analyzed by Western blot (Fig. 8B). SopB-FLAG expression was eliminated in STM transformed with plasmids encoding both RpoA negative dominants (pLAD235 and pLAD256). Controls included the *invF*::Tn5 mutant, the bacteria transformed with the empty vector (pINIIIA1) and wild type RpoA (pLAX185). The observed discrepancy between the *sopB* transcription and the translation could be explained by multiple factors including selection of normalized gene for RT-PCR, mRNA stability and translation. Despite these it is clear that *sopB* expression depends on the presence a wild type RpoA and the InvF/SicA complex. Together these results showed that α-CTD is necessary for the expression of the InvF-dependent gene *sopB*.

## Discussion

InvF is an AraC/XylS-like transcriptional regulator important for the transcription of virulence genes encoded inside and outside of SPI-1 in STM, such as *sicA* and *sopB*, among others. The products of these genes are necessary for this bacterium invasion to epithelial cells^9^. In previous reports we and others have shown that InvF acts as a monomer, that SicA is necessary to activate gene transcription, and that InvF likely functions as a classical regulator that likely recruits the transcriptional machinery to the promoter^9,10,31^. Thus, our working hypothesis here was that InvF interacts with the RNAP in a similar way as other classical bacterial transcriptional activators including a few from the AraC/XylS-like regulators^34^. In this report we have demonstrated by different approaches that InvF binds to the alpha subunit of the RNAP and that SicA is also able to independently contact both proteins.

InvF belongs, together with MxiE and BsaN, to a singular group in the AraC/XylS family of transcriptional activators as they require of small proteins to function as co-activators^34^. In these activators the need of their respective co-activator has been shown but the molecular interactions between them and the transcriptional machinery has not been shown. Previously, we demonstrated that InvF acts as a monomer and that it is required for expression of *sopB* in the absence of the repressor H-NS^10^, suggesting that it is a classical activator and as such it likely makes contacts with the RNAP. Results presented here corroborated that InvF indeed interacts with RpoA, the alpha subunit of the RNAP, confirming our hypothesis. A prediction model recently published also by our group^32^ suggests that InvF makes stable interactions with the RpoA carboxyl domain (*α*-CTD). Here, this was experimentally corroborated by using purified versions of InvF and RpoA, a bacterial two hybrid system, and with the use of RpoA negative dominant mutants. Moreover, molecular dynamics simulations between these two proteins suggests that *α*-CTD interacts with both InvF domains, the amino terminus domain (NTD) and the DNA binding domain (DBD)^32^. This was also experimentally verified here, proving again the bioinformatic predictions. Interactions of other AraC/XylS regulators with the RNAP in both domains have been shown, such as the NTD in XylS^14^, and the DBD in MelR, SoxS, MarA and Rob^13,17–22,26^. These reports indicate that these AraC/XylS regulators interact with the RNAP by the NTD or the CTD domains but interactions with both domains had not been shown as here is reported for InvF. Future experiments will include the generation of InvF point mutations in those residues likely interacting with *α*-CTD. Lastly, the bioinformatic analyses also predicted that RpoA stabilizes the DBD in InvF. Whether this stabilization changes or modifies InvF affinity for its DNA target will be experimentally tested in our laboratories.

**Figure 9:**
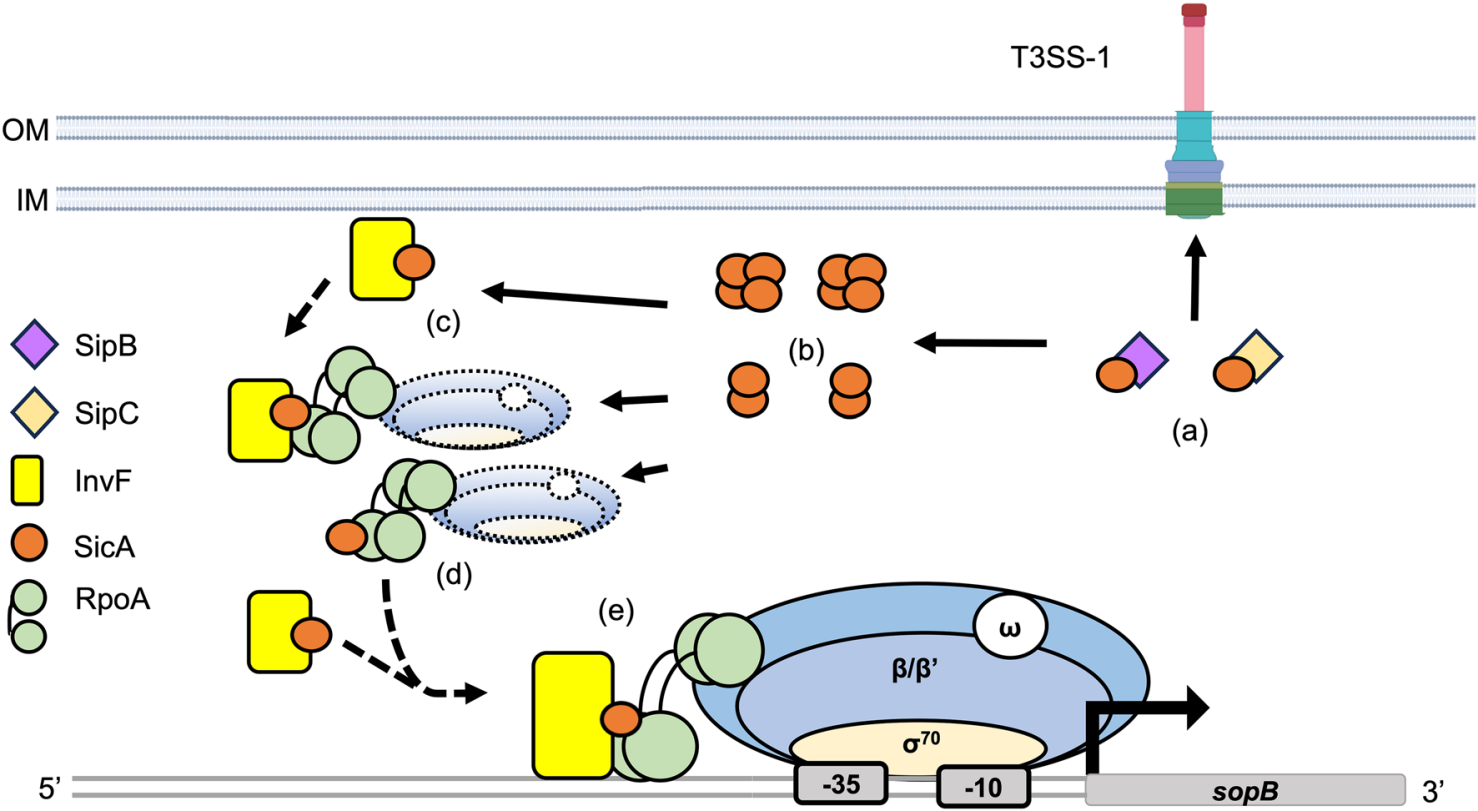
Model for the transcription of *sopB* by InvF, SicA and the RNAP*.* In this model SicA first delivers the translocators SipB and SipC to the T3SS-1 (a); then SicA is able to form dimers or tetramers (b) and is able to either interact with invF (c), RpoA (d), or both; once the InvF-SicA complex is bound to the RNAP through RpoA they bind to the *sopB* promoter region to initiate transcription. Alternatively, InvF could be recruited to the promoter region by the SicA/RNAP complex or that the InvF/SicA complex recruits the RNAP (e).

The InvF-SicA interaction and its relevance for transcriptional activation was shown since the initial studies done in Dr. Virginia Miller laboratory^9,12,31^. Recently, the InvF/SicA complex has been corroborated to be necessary for transcriptional activation^10,35,36^. Li and coworkers^35^ also showed that SicA can detect the second messenger cyclic-di-GMP (c-di-GMP) and that this molecule abolishes SicA interaction with InvF, SipB and SipC. In contrast with our previous study, they showed that SicA is necessary for InvF to interact with the DNA^10^. A possible difference is that in our study we used a fusion of InvF to the MBP while they used an His_6_-tagged version. What both studies coincide in is with the fact that the InvF/SicA complex is necessary for transcriptional activation. SicA is a class II chaperone from the CesD/SycD/LcrH family of T3SS chaperones that is involved in SipB and SipC effectors transport, in addition to its role as transcriptional co-activator making this protein one of the most indispensable for virulence in *Salmonella*^37,38^. SicA contains three tetratricopeptide repeat-like motifs (TPR) that have been shown by Kim and coworkers^36^ to be relevant for protein interactions and transcriptional activation. In this same report, they observed that point mutations in each of the three TPRs were affected in their stability when expressed from their own promoter and when over-expressed they were unable to completely activate expression of *sipB* and *sopB* (*sigD*), purified versions of these mutants were able to interact with InvF though TPR2 and TPR3 mutants had a weaker interaction. Li et al.^35^ showed that residues in these TPRs are relevant to detect c-di-GMP and that a SicA N70A mutant can contact InvF, to activate transcription but is unable to detect this second messenger. Given that the InvF/SicA complex is needed for a complete transcriptional activation it is possible that SicA has a complementary role. In silico study identified three cavities in the InvF/RNA complex and a docking study allowed us to obtain the mode of interaction between the three TPR motifs of SicA with the predicted cavities^32^. These predictions were corroborated with a molecular dynamics simulation that revealed the role of SicA in the molecular stabilization of InvF DBD motif. Although here we did not seek to determine whether the latter was occurring, results showed that RpoA, InvF and SicA form a complex in solution and that each of the proteins are able to interact with the other independently. To the best of our knowledge, this is the first report showing the interaction of a T3SS chaperone with subunits of the RNAP. These results would also explain why the TPR mutants described by Kim though interacting with InvF fail to activate transcription of *sipB* and *sopB,* probably by affecting the interaction with RpoA^36^. Future experiments need to be done to prove this possibility. In this sense, it is possible to propose that once SicA delivers the effectors SipB and SipC it can interact with either InvF or the RNAP through RpoA or with the InvF/RNAP complex, then, this oligomeric complex would be able to initiate transcription in the InvF-dependent genes (Fig. 9).

In conclusion, here we have presented evidence showing that RpoA interacts with InvF and that this contact is necessary for transcription of InvF-depending genes, such as *sopB*. Moreover, SicA, a T3SS chaperone can not only bind InvF but to also interact with RpoA, suggesting that SicA stabilizes the InvF DBD, facilitating the interaction with RpoA, and thus likely promoting transcription.

## Methods

### Bacterial strains and culture conditions

The strains used in this study are listed in Supplementary Table S1. Lysogeny–Bertani (LB) or LB-Miller broth were used for bacterial cultures at 37 °C. When indicated antibiotics were added at the following concentrations: ampicillin (100 μg/mL), streptomycin (100 μg/mL), chloramphenicol (30 μg/mL), kanamycin (30 μg/mL), and tetracycline (10 μg/mL). SPI-1 inducing conditions were as described before^16^.

### DNA manipulations

Plasmid DNA was purified using the Wizard Plus SV Minipreps DNA Purification Systems kit (Promega). The oligonucleotides used for amplification were synthetized by the IBT-UNAM and are listed in Supplementary Table S2. PCR reactions were performed by using DreamTaq Green PCR Master Mix (2X) (Thermo Fisher Scientific). Purified plasmids and PCR products were observed in 1% agarose gels stained with either ethidium bromide (Sigma) or SYBR green (Thermo Fisher Scientific).

The *sopB* gene was 3XFLAG-tagged in the *S.* Typhimurium SL1344 strain using a previously reported method based on the λRed one-step inactivation using pSUB11 plasmid as template^39^ and oligos sopBFlag-1 and sopBFlag-2 (Supplementary Table S2), generating the MD1163 (*sopB*::*3XFLAG-kan*) strain. Kanamycin resistance was removed with plasmid pCP20 as described previously to generate MD1180 (*sopB*::*3XFLAG*) strain. SopB-FLAG expression was tested by Western blot as described below.

### Construction of plasmids

Plasmids used in this work are listed in Supplementary Table S1. To construct plasmid pTOPO-SicA-FLAG, sicA-RBS-Fw and SicA-FLAGrv oligos were used to amplify *sicA* using STM genomic DNA as template, the amplicon was then cloned into pCRTOPO 2.1 using the TOPO-TA cloning kit (Thermo Fisher Scientific) following the instructions provided by the company. Plasmids pSR658-NTDInvF and pSR658-CTDInvF were obtained by amplifying the two separated domains of InvF using pSR658-InvF as template with oligos lexA-InvF-Fw, invf-NDT-Rv, CTDInvFfw and InvFrevLexA (Supplementary Table S2) and cloned into pSR658. Plasmid pSR659-RpoA was obtained by amplifying *rpoA* from pET28-RpoA with oligos RpoALexAFw and RpoALexARv and cloned into pSR659. All plasmid constructs were sequenced by Macrogen, Inc. (South Korea).

### Expression and purification of MBP-InvF and MBP

Expression and purification of MBP-InvF and MBP was done by affinity chromatography with amylose resin as described previously^10^. The proteins were dialyzed in buffer containing Tris-base 20 mM, KCl 50 mM, DTT 1 mM and glycerol 5% with a D-Tube Dialyzer Mega 20 mL MW 3.5 kDa (Millipore). The concentration of purified proteins was determined by using the BCA Protein Assay Kit (Thermo Scientific) and analyzed in a 12% sodium dodecyl sulfate-polyacrilamide (SDS-PAGE) gel electrophoresis. Aliquots were stored at − 20 °C until used.

### Expression and purification of His6-RpoA and SicA-His_6_

The His-tagged proteins were purified by affinity chromatography with Ni–NTA agarose (Qiagen) as described previously^10^. Proteins were dialyzed and protein concentration was determined as mentioned above. Aliquots were stored at − 20 °C until used.

### SicA antibodies

Polyclonal anti-SicA antibodies were produced in pathogen-free eight-week-old female BALB/c mice under standard animal facility conditions following the protocol approved by the ethics committee of the Instituto de Biotecnología UNAM (IBT- UNAM) (https://www.ibt.unam.mx/documentos/general/aprobadocombioet27nov2019protocolo-pdf-694.pdf) that complies with the guidelines described by the Canadian Council on Animal Care (https://ccac.ca/Documents/Standards/Guidelines/Antibody_production.pdf). We confirm that this study is reported in accordance with ARRIVE guidelines (https://arriveguidelines.org). Briefly, they were inoculated with 10 µg of purified protein mixed with incomplete Freund's adjuvant (Sigma-Aldrich) via intraperitoneal injection in a total volume of 200 µl. The mice were immunized 4 times at 21-day intervals. Prior to each immunization, a blood sample was collected through a small tail cut, and the serum was collected and stored at − 20 °C until analysis. A direct ELISA was conducted using the purified SicA protein as the antigen, and the titers of each obtained serum were determined. Once a maximum detection was achieved with a titer of 1:400, the mice were euthanized under general anesthesia. Blood was obtained via cardiac puncture and subsequently centrifuged to obtain the serum. The sera from three mice were collected, mixed to form a homogeneous pool, aliquoted, and stored at − 20 °C. These antibodies were tested for Western blot with purified SicA, MBP-InvF and RpoA to determine whether possible cross-reactivity.

### Pull-downs for InvF-RpoA interactions

Prior to the experiments, the expression of chimeric proteins obtained in this work were evaluated by Western blot (Fig. S1). Pull-down experiments were performed with purified MBP, MBP-InvF and His_6_-RpoA. The following mixtures were done: a negative control with MBP and His_6_-RpoA and MBP-InvF and His_6_-RpoA; mixtures were done by using 50 μg of each protein in an 2X interaction buffer (100 mM NaH_2_PO_4_, 600 mM NaCl, 40 mM imidazole, 0.5% NP-40 and 20% glycerol, pH 8.0)^40^. Proteins were let to interact for 30 min on ice, then 50 μL of amylose resin (New England Biolabs) were added to each mixture and let to interact for 2 h in agitation at ~ 4 °C in a tube rotator (Thermo Scientific). Beads were centrifuged at 2000 × g for 2 min, amylose beads were washed three times with cold washing buffer. After the last washing step, the supernatant was removed carefully and then 20 μL of Laemmli buffer were added. Samples were resolved in an 12% SDS-PAGE and stained with Coomassie blue. Western blot was performed by transferring the proteins from the SDS-PAGE to a PVDF membrane (Merck) by following a previously described protocol^10^. Western blot was developed with a His-Probe (1:5,000) (ThermoFisher) and anti-MBP antibodies (1:10,000) (New England Biolabs) by using chemiluminescence kit (Invitrogen) and observed in a Chemidoc imaging system (Biorad).

Pull-down experiments were also performed with cell-free extracts containing InvF-FLAG and His_6_-RpoA. For this, S. Typhimurium *invF::3xFLAG* transformed with pET28-RpoA and cell-free extract was obtained from bacterial cultures grown in SPI-1-inducing conditions complemented with IPTG 1 mM. Then 100 μL of the cell-free extract were mixed with 50 μL of Ni–NTA resin and let to interact for 2 h in agitation at ~ 4 °C in 1X interaction buffer. Samples were centrifuged at 2000 × g for 2 min and washed four times with low imidazole buffer. After the last washing step supernatant was removed carefully, 30 μL of Laemmli buffer were added to the beads and samples were boiled for 10 min. Samples were resolved in a 12% SDS-PAGE, Western blot was performed by transferring the proteins to a PVDF membrane and using anti-FLAG-HRP antibodies (1:5,000) (Abcam AB49763) and His-Probe-HRP (1:5,000) as suggested by the manufacturers. Membrane development was done as described above.

### Pull-down for SicA-RpoA interactions

Experiments were performed with purified His_6_-RpoA and extract of the *E. coli* BL21 pTOPO-SicA-FLAG strain. For this 100 μL of the cell-free extract were mixed with 50 μL of purified His_6_-RpoA, 50 μL of Ni–NTA magnetic beads (Thermo Scientific) and 1X interaction buffer and let to interact for 2 h in agitation at ~ 4 °C. Samples were collected with a magnetic stand (New England Biolabs) for 2 min and washed four times with low imidazole buffer. After the last washing step supernatant was removed carefully, 30 μL of Laemmli buffer were added to the beads and samples were boiled for 10 min. Samples were resolved in a 12% SDS-PAGE, Western blot was performed by transferring the proteins to a PVDF membrane and using anti-FLAG-HRP antibodies (1:5,000) (Abcam AB49763) and HisProbe-HRP (1:5,000) as suggested by the manufacturers. Membrane development was done as described above.

### Pull-down for InvF-SicA-RpoA interactions

For the triple interaction 100 μL of cell-free extract of STM *invF::3xFLAG* transformed with plasmid pET28-RpoA were mixed with 50 μL of Ni–NTA magnetic beads and let to interact for 2 h in agitation at ~ 4 °C in 1 × interaction buffer. Samples were collected with a magnetic stand for 2 min and washed four times with low imidazole buffer. After the last washing step supernatant was removed carefully, 30 μL of Laemmli buffer were added to the beads and samples were boiled for 10 min. Samples were resolved in a 12% SDS-PAGE, Western blot was performed by transferring the proteins to a PVDF membrane and using anti-FLAG-HRP (1:5,000) and anti-SicA antibodies (1:5,000) obtained for this work (Fig. S2) and His-Probe-HRP (1:5,000) as suggested by the manufacturers. Membrane developing was done as described above.

### Interaction between InvF and STM cell-free extracts

In order to corroborate interactions of InvF with cytoplasmic proteins from STM, cell free extracts of STM WT and STM *invF::Tn5* were used. First 70 μL of amylose magnetic beads (New England Biolabs) previously washed with a washing buffer were mixed with MBP-InvF 32.5 μg/μL or MBP 10 μg/μL as a control and let to interact overnight at 4 °C. Then the beads were washed three times with 200 μL washing buffer and 400 μL of cell-free extracts were added and let to interact in agitation at 4 °C for 6 h. After the incubation the beads were washed 10 times with a washing buffer. After the last washing step supernatant was removed, 30 μL of Laemmli buffer were added to the beads and samples were boiled for 10 min. Samples were resolved in a 12% SDS-PAGE. Differential bands were cut from the gel and the proteins were characterized by LC/MS–MS at the Proteomics Discovery Platform of the Institut de Recherches Cliniques de Montréal (Quebec, Canada). Scaffold (version Scaffold_5.2.0, Proteome Software Inc., Portland, OR) was used to validate MS/MS based peptide and protein identifications. Peptide identifications were accepted only if they could be established at greater than 95.0% probability by the Scaffold Local FDR algorithm. Protein identifications were accepted only if they could be established at greater than 95.0% probability and contained at least 2 identified peptides. Protein probabilities were assigned by the Protein Prophet algorithm^41^. Proteins that contained similar peptides and could not be differentiated based on MS/MS analysis alone were grouped to satisfy the principles of parsimony.

### Dimerization assays

A LexA-based two hybrid system was used to evaluate protein–protein interactions between InvF, SicA and RpoA^27,28^. To verify the integrity of the LexA-derived proteins a Western blot was performed by using the anti-LexA antibody (Millipore) (1:5,000) and recombinant protein G-HRP (Thermo Scientific) (1:10,000) (Fig. S3). To test InvF and SicA heterodimerization competent cells of *E. coli* SU202 were transformed individually or combined with pSR658-InvF, pSR658-NTDInvF, pSR658-CTDInvF, pSR659-SicA and pSR659-RpoA, and selected in LB plates with the corresponding antibiotics. Transformants were grown in LB supplemented with 1 mM IPTG and let them grow to an OD_600_ = 0.6. Aliquots were taken to assess β-galactosidase activity as described before^10^. Plasmids pSR658, pSR659, pSR658-HilD and pSR659-HilE were used as controls.

### RT-qPCR assays

Relative expression of *sopB* in the different STM strains was determined by RT-qPCR as described previously^10^. Briefly, RNA was obtained from bacterial cultures grown in SPI-1-inducing conditions. DNA was removed with DNA-Free (Ambion) and then cDNA was obtained with a GoScript kit (Promega). qPCR was performed in a Rotor gene Q Thermocycler (Qiagen). Relative expression of *sopB* was calculated with the ^ΔΔ^Ct method using the expression of the gene *gyrB* as a normalizer. Oligos for each gene are listed in Supplementary Table S2. Experiments were done in triplicates and the results are the average of three independent experiments.

### *sopB* expression assay

Briefly, 350 μL of an overnight culture of STM *sopB::FLAG*, STM *invF::Tn5 sopB::FLAG* and STM *sopB::FLAG* transformed with pINIIIA1, pLAX185, pLAD235 and pLAD256 were inoculated in flasks with 10 mL of LB-Miller complemented with IPTG 1 mM, the cultures were incubated for 3.5 h at 37 °C in agitation (225 rpm) and the optical density was measured at 600 nm. The cultures were centrifugated for 20 min at 11,000 × g at 4 °C, the supernatants were discarded, then 100 μL of Laemmli buffer were added to the pellets and were heated at 95 °C for 10 min. Samples were loaded onto a 12% SDS-PAGE and stained with Coomassie stain. Western blot was performed by transferring the proteins to a PVDF membrane (Merck) and using anti-FLAG-HRP (1:5,000), anti-GroEL(1:10,000) antibodies (Abcam) and recombinant protein G-HRP. Membrane development was done as described above.

### Statistical analysis

Statistical analysis was performed in GraphPad Prism version 6.01 (Graph-Pad Software) by using a Student’s *t*-test. A significant difference was considered when *P* < 0.01 for the dimerization assays and 0.05 for the RT-qPCR assays.

### Supplementary Information

Supplementary Information.

## Data Availability

All data generated or analysed during this study are included in this published article [and its supplementary information files].
