# Supplementary material for: Interactions between the AraC/XylS-like transcriptional activator InvF of Salmonella Typhimurium, the RNA polymerase alpha subunit and the chaperone SicA
Source: Sci Rep. 2024 Jan 2;14:156. doi: 10.1038/s41598-023-50636-w (PMC10761746; doi:10.1038/s41598-023-50636-w)
Supplement: Supplementary file 1 — Supplementary Information. [file 41598_2023_50636_MOESM1_ESM.pdf]

Supplementary material for the manuscript:

Interactions between the AraC/XylS-like transcriptional activator InvF of *Salmonella* Typhimurium, the RNA polymerase alpha subunit and the chaperone SicA

Daniel Cortés-Avalos, André Borges Farias, Luis E. Romero-González, Cristina Lara-Ochoa, Lourdes Villa-Tanaca, Francisco García-del Portillo, Vanessa López-Guerrero, Víctor H. Bustamante, Ernesto Pérez-Rueda and J. Antonio Ibarra

Supplementary Figure 1

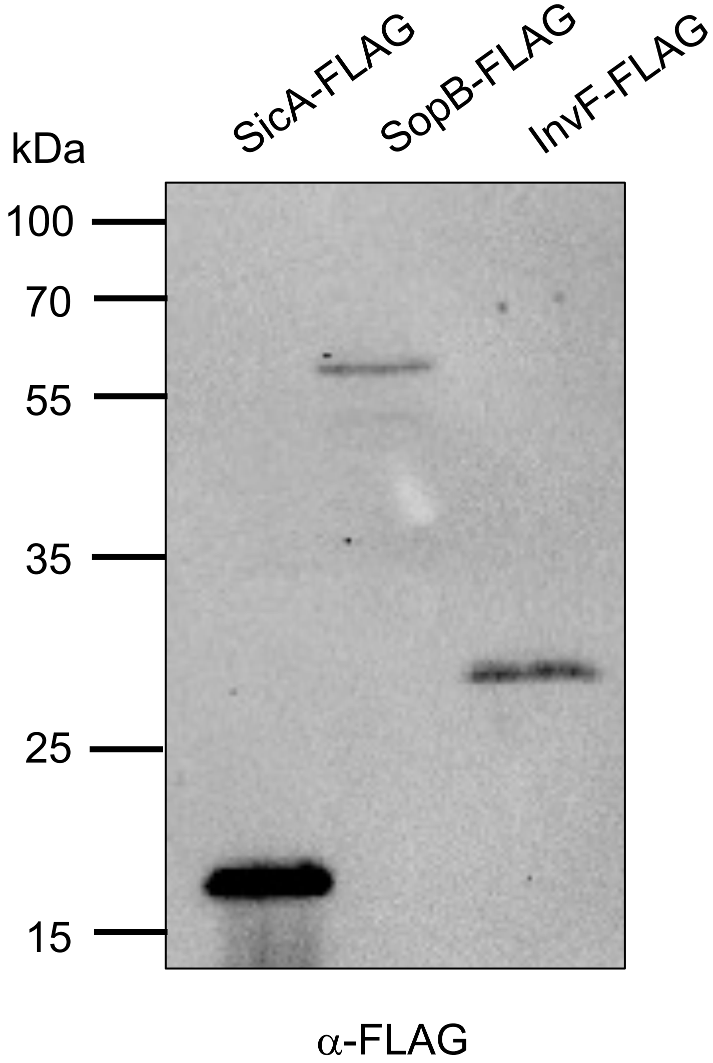

Supplementary figure S1. Expression of FLAG-fused proteins. Proteins were overexpressed with IPTG 1 mM and identified by Western blot with anti-FLAG-HRP as described in the methods section.

Supplementary Figure 2

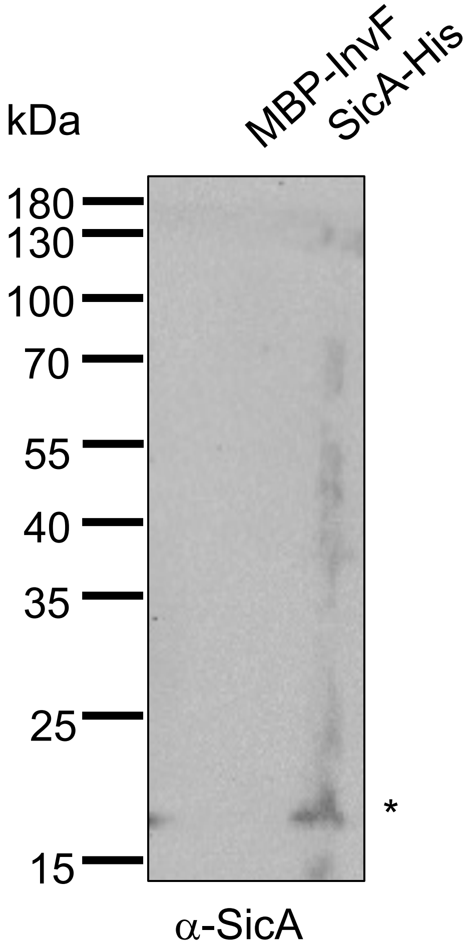

Supplementary figure S2. Characterization of anti-SicA antibodies. Purified SicA-His6 protein was used to generate antibodies in mice. Purified proteins His6-RpoA, MBP-InvF and SicA-His6 were used to test mouse polyclonal anti-SicA antibodies by Western blot. Recombinant protein G-HRP was used as a secondary antibody.

Supplementary Figure 3

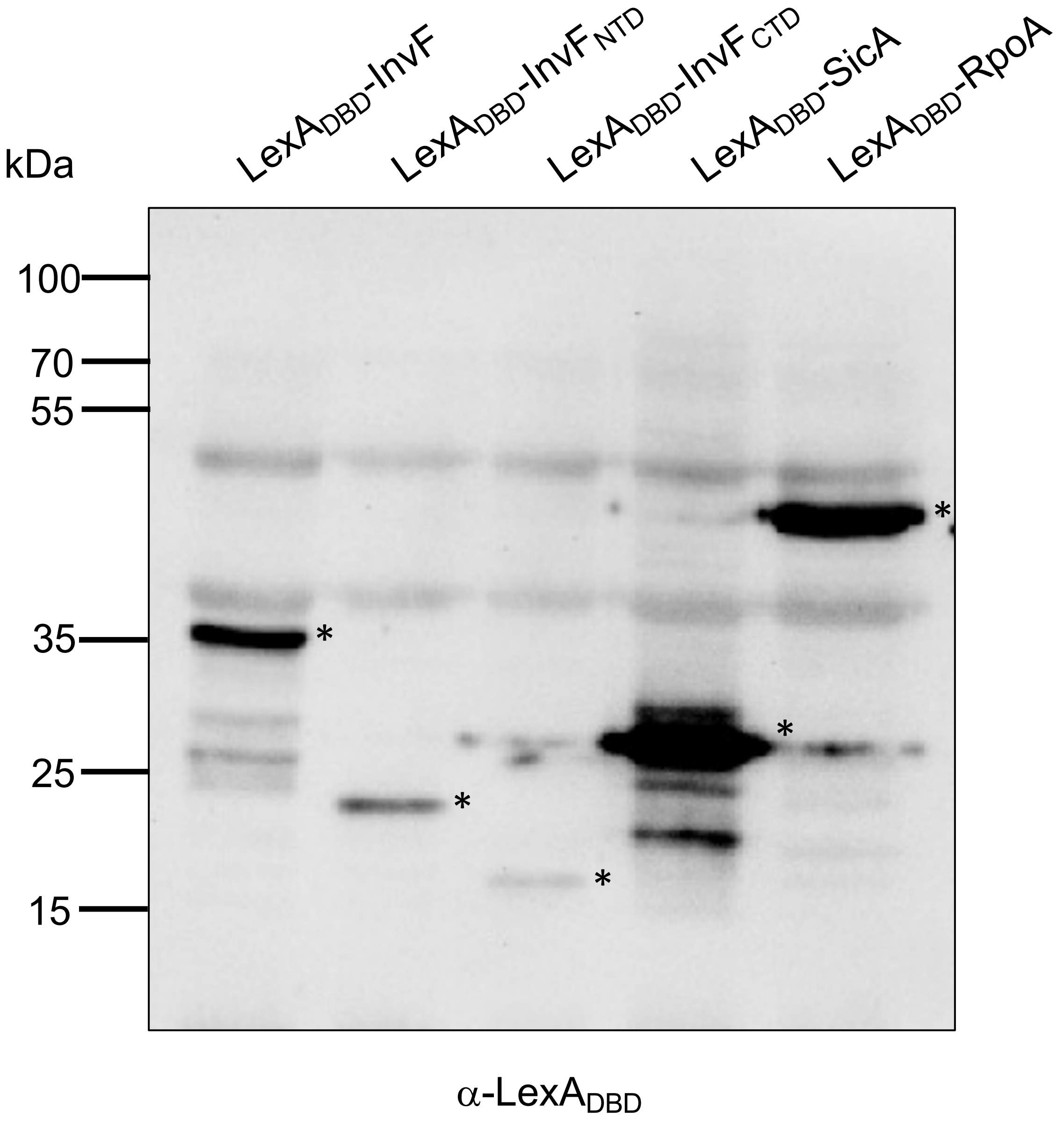

Supplementary figure S3. Expression of LexA-fused proteins. Proteins were overexpressed with IPTG 1 mM and identified by Western blot with anti-LexA antibodies and recombinant protein G-HRP.

Supplementary table S1. Strains and plasmids used in this work.

**Strain or plasmid**	**Genotype or description**	**Reference[a]**
Salmonella Typhimurium strains		
SL1344	Wild-type SL1344, Sm^R	[1]
SL1344 ΔSPI-1	Wild-type SL1344 isogenic mutant, Km^R	[2]
SL1344 *invF*::*Tn5*	SL1344 *invF::Tn5* mutant, Km^R	[3]
SL1344 *invF*::*Tn5* *sopB::FLAG*	Obteined by transducing *invF*::*Tn5* from SL1344 *invF*::*Tn5* into SL1344 *sopB::FLAG* Km^R	This work
SL1344 *invF::3xFLAG-kan*	Obtained by transducing *invF::3xFLAG-kan* from *Salmonella* strain 14028 into SL1344, Km^R	[4]
SL1344 *invF::3xFLAG*	Derivative SL1344 *invF::3xFLAG-kan,* Sm^R	[4]
MD1163	SL1344 *sopB::3xFLAG-Km*	This work
MD1180	SL1344 *sopB::3xFLAG*	This work
Escherichia coli strains		
DH10B	Laboratory cloning strain, Sm^R	Invitrogen
BL21	Strain for expression of recombinant proteins	Invitrogen
SU202	Reporter strain harboring a *sulA::lacZ* transcriptional fusion	[5]
Plasmids		
pMal-InvF	pMAL-c2X derivative expressing MBP-InvF from a *tac* promoter, Ap^R	[6]
pMal-c2xa	Low copy number plasmid to generate fusions to the *malE* gene for expressing MBP, Ap^R	New England Biolabs
pTOPO-SicA	pCRTOPO derivative expressing SicA-His_6 from the T7 promoter, Km^R Ap^R	[6]
pET28-RpoA	pET28a derivate expressing His_6-RpoA, Km^R	[7]
pINIIIA1	pBR322 derivate cloning vector for mutants of *rpoA*, Ap^R	[8]
pLAX185	pINIIIA1derivate expressing wild type RpoA, Ap^R	[9]
pLAD235	pINIIIA1derivate expressing RpoA truncated from the carboxy end, which retain 235 amino acids, Ap^R	[9]
pLAD256	pINIIIA1derivate expressing RpoA truncated from the carboxy end, which retain 256 amino acids, Ap^R	[9]
pTOPO-SicA-FLAG	pCRTOPO derivative expressing SicA-FLAG from the T7 promoter, Km^R Ap^R	This work

pSR658	ColE1 derivative cloning vector, *lac* promoter, Tc^R	[10]
pSR659	p15A derivative low-copy-number cloning vector, *lac* promoter, Ap^R	[10]
pSR658-InvF	pSR658 derivative expressing $LexA_{DBDwt}$-InvF from the *lac* promoter	[6]
pSR658-SicA	pSR658 derivative expressing $LexA_{DBDwt}$-SicA from the *lac* promoter	[6]
pSR658-NTDInvF	pSR658 derivative expressing $LexA_{DBDwt}$-$InvF_{NTD}$ from the *lac* promoter	This work
pSR658-CTDInvF	pSR658 derivative expressing $LexA_{DBDwt}$-$InvF_{CTD}$ from the *lac* promoter	This work
pSR659-SicA	pSR659 derivative expressing $LexA_{DBDmut}$-SicA from the *lac* promoter	[6]
pSR659-RpoA	pSR659 derivative expressing $LexA_{DBDmut}$-RpoA from the *lac* promoter	This work
pSR658-HilD	pSR658 derivative expressing $LexA_{DBDwt}$-HilD from the *lac* promoter	[11]
pSR659-HilE	pSR659 derivative expressing $LexA_{DBDmut}$-HilE from the *lac* promoter	[11]

[a.] **References for Supplementary table 1.**

1. Hoiseth, S.K. & Stocker, B.A. Aromatic-dependent *Salmonella typhimurium* are non-virulent and effective as live vaccines. *Nature*. **21**, 8-9; 10.1038/291238a0 (1981).

2. Drecktrah, D., Knodler, L.A., Ireland, R. & Steele-Mortimer, O. The mechanism of *Salmonella* entry determines the vacuolar environment and intracellular gene expression. *Traffic*. **7**, 39-51; 10.1111/j.1600-0854.2005.00360.x (2006).

3. Kaniga, K., Bossio, J.C. & Galán, J.E. The *Salmonella typhimurium* invasion genes *invF* and *invG* encode homologues of the AraC and PulD family of proteins. *Mol. Microbiol.* **13**, 55-68; 10.1111/j.1365-2958.1994.tb00450.x (1994).

4. De la Cruz, M.A. et al. The two-component system CpxR/A represses the expression of *Salmonella* virulence genes by affecting the stability of the transcriptional regulator HilD. *Front Microbiol.* **6**, 807; 10.3389/fmicb.2015.00807 (2015).

5. Dmitrova, M. et al. A new LexA-based genetic system for monitoring and analyzing protein heterodimerization in *Escherichia coli*. *Mol. Gen. Genet.* **257**, 5-12; 10.1007/s004380050640 (1998).

6. Romero-González, L.E. et al. The *Salmonella* Typhimurium InvF-SicA complex is necessary for the transcription of *sopB* in the absence of the repressor H-NS. *PLoS One.* **15**, e0240617; 10.1371/journal.pone.0240617 (2020).

7. Rebollar-Flores, J. E. et al. The *Salmonella enterica* serovar Typhi *ltrR* gene encodes two proteins whose transcriptional expression is up-regulated by alkaline pH and repressed at their promoters and coding regions by H-NS and Lrp. *J. Bacteriol.* **202**, e00783-e819; 10.1128/JB.00783-19 (2020).

8. Masui,Y., Mizuno, T. & Inouye, M. Novel high-level expression cloning vehicles: 104-fold amplification of *Escherichia coli* minor protein. *Nat. Biotechnol.* **2**, 81–85; 10.1038/nbt0184-81 (1984).

9. Hayward, R.S., Igarashi, K. & Ishihama, A. Functional specialization within the alpha-subunit of *Escherichia coli* RNA polymerase. *J. Mol. Biol.* **221**, 3-9; 10.1016/0022-2836(91)80197-3 (1991).

10. Daines, D.A. & Silver, R.P. Evidence for multimerization of *neu* proteins involved in polysialic acid synthesis in *Escherichia coli* K1 using improved LexA-based vectors. *J. Bacteriol.* **182**, 67-70; 10.1128/JB.182.18.5267-5270.2000 (2000).

11. Paredes-Amaya, C.C., Valdés-García, G., Juárez-González, V.R., Rudiño-Piñera, E. & Bustamante, V.H. The Hcp-like protein HilE inhibits homodimerization and DNA binding of the virulence-associated transcriptional regulator HilD in *Salmonella*. *J. Biol. Chem.* **293**, 6578-6592; 10.1074/jbc.RA117.001421(2018).

Supplementary table S2. Oligonucleotides used in this work.

Primer name	Sequence (5'-3)'	Tm (°C)
sicA-RBS-Fw	CTCGAGATTAAAGGAGGTAAATAATGGATTATCAAAAT AATGTCAGCG	53
SicA-FLAGrv	TTACTTGTCGTCATCGTCTTTGTAGTCTTCCTTTTCTTG TTCACTGTGC	53
RpoALexAFw	CCGCTCGAGATGCAGGGTTCTGTGAC	52
RpoALexA Rv	GGGGTACTTAGTCGTCAGCGATGC	52
invf-NDT-Rv	GTCTCTAGATCAGAAGGCCGGAGAAGGCGT	60
lexA-InvF-Fw	CCGCTCGAGATGCTAAATACGCAGGAAGTAC	52
CTDInvFfw	CCGCTCGAGAATAAGGTACTGGCGCTG	52
InvFrevLexA	GGGGTACCTCATTTGTCTGCCAATTG	48
sopB-RT-Fw	AAGCAGCTTAATAACCAGCCC	55
sopB-RT-Rv	ACCGTCCTCATGCACACTCAC	59
sopBFlag-1	TTGGCAGTCAGTAAAAGGCATTTCTTCATTAATCACATC TGACTACAAAGACCATGACGG	
sopBFlag-2	TAAACGATTTAATAGACTTTCCATATAGTTACCTCAAGA CCATATGAATATCCTCCTTAG	
gyrB-Fw	TGGTTTCCCACAGCTGATCC	59
gyrB-Rv	TTCAATCAGACCACGCAGCT	58

Supplementary file 1

Jose_20180130_Refseq_Salmonella_Typhimurium_20180219_InvF-Pulldown_SCAFF4, Samples report created on 07/27/2023
Experiment: Jose_20180130_Refseq_Salmonella_Typhimurium_20180219_InvF-Pulldown_SCAFF4

- Peak List Generator: unknown
  - Version: unknown
  - Charge States Calculated: unknown
  - Deisotoped: unknown
  - Textual Annotation: unknown
- Database Set: 1 Database
  - Database Name: Refseq_Salmonella_Typhimurium
    - Version: unknown
    - Taxonomy: All Entries
    - Number of Proteins: 0
  - Explain Database w/ < 1000 entries:
  - Does database contain common contaminants?: unknown
- Search Engine Set: 1 Search Engine
  - Search Engine: Mascot (Ion Score Only)
    - Version: Mascot in Proteome Discoverer 2.1.0.81
    - Samples: All Samples
    - Fragment Tolerance: 0.60 Da (Monoisotopic)
    - Parent Tolerance: 10.0 PPM (Monoisotopic)
    - Fixed Modifications: +57 on C (Carbamidomethyl)
    - Variable Modifications: +16 on M (Oxidation)
    - Database: Refseq_Salmonella_Typhimurium (unknown version, 0 entries)
    - Digestion Enzyme: Trypsin
    - Max Missed Cleavages: 2
    - Probability Model:
      - VL_20180216_ADH_37: Peptide Prophet with Delta Mass Correction [+2 and below,+3,+4,+5,+6,+7,+8,+9,+10,+11,+12,+13,+14,+15,+16,+17,+18,+19,+20,+21,+25 and above]
      - VL_20180219_IBA_01: Peptide Prophet with Delta Mass Correction [+2 and below,+3,+4,+5,+6,+7,+8,+10,+12,+19 and above]
      - VL_20180219_ADH_01: Peptide Prophet with Delta Mass Correction [+2 and below,+3,+4,+5,+6,+7,+8,+11,+12,+13,+14,+15,+16,+17,+18,+19,+20,+21,+23,+24 and above]
      - VL_20180219_IBA_02: Peptide Prophet with Delta Mass Correction [+2 and below,+3,+4,+5,+6,+7,+8,+9,+10,+11,+12,+13,+16,+17,+18,+19,+21 and above]
      - VL_20180219_ADH_02: Peptide Prophet with Delta Mass Correction [+2 and below,+3,+4,+5,+6,+7,+8,+9,+10,+11,+12,+13,+14,+16,+17,+19,+20,+21 and above]
      - VL_20180219_IBA_03: Peptide Prophet with Delta Mass Correction [+2 and below,+3,+4,+5,+6,+7,+8,+9,+10,+12,+13,+14,+15,+16,+17,+18,+19,+22 and above]
      - VL_20180219_ADH_04_2: Peptide Prophet with Delta Mass Correction [+2 and below,+3,+4,+5,+6,+7,+9,+10,+11,+12,+13,+14,+15,+16,+17,+18,+19,+20,+21,+23 and above]
      - VL_20180219_IBA_04: Peptide Prophet with Delta Mass Correction [+2 and below,+3,+4,+5,+6,+7,+8,+10,+11 and above]
      - VL_20180219_ADH_05: Peptide Prophet with Delta Mass Correction [+2 and below,+3,+4,+5,+6,+7,+8,+10,+11,+13,+15,+16,+17,+18,+19,+20,+21,+22 and above]
      - VL_20180219_IBA_05: Peptide Prophet with Delta Mass Correction [+2 and below,+3,+4,+5,+6,+7,+8,+9,+11 and above]
      - VL_20180219_ADH_06_2: Peptide Prophet with Delta Mass Correction [+2 and below,+3,+4,+5,+6,+7,+8,+10,+11,+12,+13,+14,+15,+17,+19,+20,+21 and above]
      - VL_20180219_IBA_06: Peptide Prophet with Delta Mass Correction [+2 and below,+3,+4,+5,+6,+7,+8,+9,+10,+11,+12,+22 and above]
- Scaffold: Version: Scaffold_5.2.0
  - Modification Metadata Set: 2334 modifications
    - Source: C:\Program Files\Scaffold 4\parameters\unimod.xml
    - Comment:
  - Protein Grouping Strategy: Experiment-wide grouping with protein cluster analysis
  - Peptide Thresholds: 95.0?% minimum
  - Protein Thresholds: 95.0?% minimum and 2 peptides minimum
  - Peptide FDR: 0.0?% (Decoy)
  - Protein FDR: 0.0?% (Decoy)
  - GO Annotation Source(s):
  - Pathway Annotation Source(s): Unknown
  - Alternate ID Source(s):

Jose_20180130_Refseq_Salmonella_Typhimurium_20180219_InvF-Pulldown_SCAFF4								Stimated size (kDa)		40		50		100		15		50		70
#	Visible?	Starred?	Identified Pr	Accession Nu	Alternate ID	Molecular W	Protein Grou	Taxonomy	00-BLK befor	01-Band 5	02-BLK befor	03-Band 4	04-BLK befor	05-Band 1	06-BLK befor	07-Band 6	08-BLK befor	09-Band 3	10-BLK befor	11-Band 2
1	true	Empty	GroEL protei	CBW20353.1		57 kDa		unknown	0	79	14	182	25	35	0	0	0	1633	280	79
2	true	Empty	glycerol kina	CBW20124.1		56 kDa		unknown	0	62	4	644	210	36	43	12	23	15	7	22
3	true	Empty	periplasmic	CBW20253.1 (+1)		43 kDa		unknown	0	120	57	78	23	53	7	4	2	67	22	124
4	true	Empty	MULTISPECIE	WP_000301869.1		30 kDa		unknown	0	88	14	65	9	61	3	30	4	45	4	52
5	true	Empty	AraC family	WP_001674874.1		24 kDa		unknown	0	15	5	10	8	38	2	3	4	54	16	244
6	true	Empty	MULTISPECIE	WP_001216370.1		14 kDa		unknown	0	4	0	0	0	4	0	247	24	2	10	2
7	true	Empty	30S ribosom	CBW19412.1		15 kDa		unknown	0	2	0	1	0	2	0	197	56	2	21	2
8	true	Empty	50S ribosom	CBW17367.1		13 kDa		unknown	0	5	2	0	0	2	0	169	89	3	34	2

9	true	Empty	Cluster of fla	WP_000079805.1 [2]	52 kDa	true	unknown	0	73	12	156	43	10	8	4	6	4	1	6
9.1	true	Empty	flagellin Fl	WP_000079805.1	52 kDa	true	unknown	0	26	2	46	9	3	1	1	0	0	0	0
9.2	true	Empty	flagellin Fl	WP_000079794.1	53 kDa	true	unknown	0	2	0	17	0	0	0	0	0	0	0	0
10	true	Empty	30S ribosom	CBW19510.1	14 kDa		unknown	0	1	0	0	0	2	0	185	13	0	7	2
11	true	Empty	chaperone pı	WP_001235094.1	95 kDa		unknown	0	0	0	0	0	217	2	0	1	0	2	54
12	true	Empty	30S ribosom	WP_000140324.1	61 kDa		unknown	0	0	0	0	0	0	0	0	0	1	0	150
13	true	Empty	elongation fa	CBW19508.1	78 kDa		unknown	0	0	0	4	0	183	3	0	0	1	0	8
14	true	Empty	glycerol-3-pł	WP_000448179.1	57 kDa		unknown	0	2	0	116	13	19	0	0	0	29	0	5
15	true	Empty	maltodextrin	WP_000082246.1	90 kDa		unknown	0	0	0	0	0	148	5	0	0	0	0	13
16	true	Empty	elongation fa	CBW20173.1	43 kDa		unknown	0	99	13	10	0	10	0	0	0	4	0	7
17	true	Empty	SspC [Salmo	AAC43547.1	43 kDa		unknown	0	129	14	0	0	0	0	0	0	0	0	0
18	true	Empty	ribonuclease	CBW20387.1	92 kDa		unknown	0	5	0	1	0	50	0	0	0	9	0	52
19	true	Empty	RecA protein	CBW18907.1	38 kDa		unknown	0	83	21	2	2	0	0	0	0	0	0	0
20	true	Empty	heat shock p	CBW16580.1 (+1)	71 kDa		unknown	0	0	0	0	0	0	0	0	0	0	0	87
21	true	Empty	DNA-directe	CBW20181.1	155 kDa		unknown	0	1	0	0	0	49	0	0	0	3	0	34
22	true	Empty	DNA gyrase s	WP_000072047.1	90 kDa		unknown	0	0	0	0	0	85	0	0	0	0	0	3
23	true	Empty	MULTISPECIE	WP_001216673.1	15 kDa		unknown	0	0	0	0	0	0	0	61	17	3	2	0
24	true	Empty	50S ribosom	CBW19495.1	15 kDa		unknown	0	3	0	0	0	4	0	66	11	0	1	0
25	true	Empty	ADP-forming	WP_001048589.1	41 kDa		unknown	0	70	5	0	0	0	0	0	0	0	0	0
26	true	Empty	dihydrolipoa	CBW16257.1	51 kDa		unknown	0	0	0	73	5	0	0	0	0	0	0	0
27	true	Empty	ATP-depende	WP_000934063.1	84 kDa		unknown	0	0	0	0	0	66	0	0	0	0	0	3
28	true	Empty	30S ribosom	CBW19478.1	23 kDa		unknown	0	14	1	5	0	12	0	19	0	6	0	1
29	true	Empty	MULTISPECIE	WP_001238917.1	15 kDa		unknown	0	0	0	2	0	2	0	50	8	1	2	3
30	true	Empty	ATP synthase	CBW19925.1	55 kDa		unknown	0	3	0	56	3	0	0	0	0	0	0	0
31	true	Empty	MULTISPECIE	WP_001029758.1	14 kDa		unknown	0	0	0	0	0	0	0	48	7	0	0	0
32	true	Empty	molecular ch	WP_000516125.1	69 kDa		unknown	0	0	0	0	0	0	0	0	0	0	0	58
33	true	Empty	trigger facto	WP_001198406.1	48 kDa		unknown	0	0	0	58	0	0	0	0	0	0	0	0
34	true	Empty	MULTISPECIE	WP_001518010.1	74 kDa		unknown	0	0	0	0	0	0	0	0	0	0	0	58
35	true	Empty	phosphoenol	CBW17380.2 (+1)	89 kDa		unknown	0	0	0	0	0	55	0	0	0	0	0	3
36	true	Empty	30S ribosom	CBW19496.1	26 kDa		unknown	0	10	1	5	1	16	0	2	0	10	0	7
37	true	Empty	MULTISPECIE	WP_000940121.1	18 kDa		unknown	0	0	0	0	0	0	0	47	6	0	0	0
38	true	Empty	Glu/Leu/Phe	WP_001526439.1	46 kDa		unknown	0	51	1	0	0	0	0	0	0	0	0	0
39	true	Empty	phosphate a	CBW18409.1	77 kDa		unknown	0	0	0	0	0	42	1	0	0	0	0	6
40	true	Empty	30S ribosom	CBW19480.1	13 kDa		unknown	0	0	0	0	0	2	0	44	3	0	0	0
41	true	Empty	DNA-directe	WP_000263106.1	151 kDa		unknown	0	0	0	0	0	34	0	0	0	0	0	14
42	true	Empty	50S ribosom	CBW20178.1	18 kDa		unknown	0	0	0	0	0	0	0	40	1	0	0	0
43	true	Empty	MULTISPECIE	WP_001138042.1	18 kDa		unknown	0	1	0	0	0	0	0	43	1	0	0	0
44	true	Empty	ribonucleosic	WP_001076487.1	86 kDa		unknown	0	0	0	0	0	37	0	0	0	0	0	0
45	true	Empty	50S ribosom	CBW19501.1	22 kDa		unknown	0	0	0	0	0	1	0	33	3	0	0	0
46	true	Empty	ATP-depende	WP_000219174.1	50 kDa		unknown	0	0	0	3	0	13	0	0	0	13	0	12
47	true	Empty	MULTISPECIE	WP_000210741.1	17 kDa		unknown	0	0	0	0	0	0	0	34	3	2	0	0
48	true	Empty	phosphoenol	CBW20157.1	99 kDa		unknown	0	0	0	0	0	34	0	0	0	0	0	0
49	true	Empty	MULTISPECIE	WP_001162094.1	37 kDa		unknown	0	34	3	0	0	0	0	0	0	0	0	0
50	true	Empty	succinate del	WP_000775561.1	64 kDa		unknown	0	3	0	4	0	0	0	0	0	6	0	22
51	true	Empty	anaerobic gly	CBW18357.1	44 kDa		unknown	0	27	0	0	0	0	0	0	0	0	0	0
52	true	Empty	polynucleotic	CBW19351.1	77 kDa		unknown	0	0	0	0	0	35	0	0	0	0	0	1
53	true	Empty	translation ir	WP_000133064.1	97 kDa		unknown	0	0	1	0	1	21	3	1	2	0	2	5
54	true	Empty	IscS subfami	WP_000775266.1	45 kDa		unknown	0	31	4	0	0	0	0	0	0	0	0	0
55	true	Empty	Threonyl-tRN	CBW17364.1	74 kDa		unknown	0	0	0	1	0	3	0	0	0	0	0	22
56	true	Empty	signal recogr	WP_000460052.1	50 kDa		unknown	0	0	0	23	1	0	0	0	0	0	0	0
57	true	Empty	transcription	CBW19966.1	47 kDa		unknown	0	17	1	4	2	1	0	0	1	0	1	1
58	true	Empty	dihydrolipoa	CBW16817.1	44 kDa		unknown	0	0	0	30	1	0	0	0	0	0	0	0
59	true	Empty	5-methyltetr	WP_000154192.1	85 kDa		unknown	0	0	0	0	0	25	2	0	0	0	0	0
60	true	Empty	lipopolysacc	CBW19782.1	42 kDa		unknown	0	19	0	0	0	0	0	0	0	0	0	0
61	true	Empty	chemotaxis p	WP_000147295.1	18 kDa		unknown	0	0	0	0	0	0	0	20	7	0	0	0
62	true	Empty	MULTISPECIE	WP_000908562.1	19 kDa		unknown	0	0	0	0	0	0	0	21	5	0	0	0
63	true	Empty	MULTISPECIE	WP_001107481.1	71 kDa		unknown	0	1	0	2	0	0	0	0	0	3	0	23
64	true	Empty	RNA polyme	CBW19283.1	71 kDa	true	unknown	0	0	0	0	0	25	0	0	0	0	0	0
65	true	Empty	glycine--tRN	WP_001291736.1	76 kDa		unknown	0	0	0	0	0	0	0	0	0	0	0	22
66	true	Empty	tryptophan s	CBW17752.1	43 kDa		unknown	0	17	0	0	0	0	0	0	0	0	0	0
67	true	Empty	outer memb	WP_001240935.1	90 kDa		unknown	0	0	0	0	0	20	0	0	0	0	0	0
68	true	Empty	energy-depe	WP_000046770.1	62 kDa		unknown	0	0	0	0	0	0	0	0	0	24	0	0

69	true	Empty	asparagine--	WP_000117870.1	53 kDa	unknown	0	0	0	21	1	0	0	0	0	0	0	0
70	true	Empty	polynucleotic	WP_000174614.1	55 kDa	unknown	0	0	0	0	0	8	0	0	0	0	0	11
71	true	Empty	fructose-bisp	WP_001250625.1	36 kDa	unknown	0	23	0	0	0	0	0	0	0	0	0	0
72	true	Empty	2-amino-3-k	CBW19769.1	43 kDa	unknown	0	21	0	0	0	0	0	0	0	0	0	0
73	true	Empty	50s ribosom	CBW20413.1	16 kDa	unknown	0	0	0	0	0	0	0	21	2	0	0	0
74	true	Empty	membrane-b	WP_000829731.1	86 kDa	unknown	0	0	0	0	0	19	0	0	0	0	0	0
75	true	Empty	anaerobic gl	CBW18355.1	59 kDa	unknown	0	0	0	0	0	0	0	0	0	18	0	0
76	true	Empty	chaperone p	WP_000758966.1	18 kDa	unknown	0	0	0	0	0	0	0	17	2	0	0	0
77	true	Empty	D-alanine ca	CBW16725.1	44 kDa	unknown	0	19	0	0	0	0	0	0	0	0	0	0
78	true	Empty	MULTISPECIE	WP_000447529.1	12 kDa	unknown	0	0	0	0	0	0	0	15	0	1	0	0
79	true	Empty	hypothetical	CBW18440.1	36 kDa	unknown	0	8	4	1	0	0	0	0	0	0	0	0
80	true	Empty	sulphate tra	CBW18506.1	41 kDa	unknown	0	16	0	0	0	0	0	0	0	0	0	0
81	true	Empty	GTP-binding	CBW20045.1	67 kDa	unknown	0	0	0	0	0	0	0	0	0	0	0	17
82	true	Empty	tRNA uridine	WP_000499872.1	70 kDa	unknown	0	0	0	0	0	0	0	0	0	0	0	10
83	true	Empty	50S ribosom	WP_000579838.1	22 kDa	unknown	0	0	0	0	0	2	0	14	0	0	0	0
84	true	Empty	glutamate 1-	CAC03102.1	45 kDa	unknown	0	15	1	0	0	0	0	0	0	0	0	0
85	true	Empty	hypothetical	CBW19566.1 (+1)	85 kDa	unknown	0	0	0	0	0	15	0	0	0	0	0	0
86	true	Empty	major outer	CBW17407.1	8 kDa	unknown	0	8	0	2	0	2	0	0	0	1	0	0
87	true	Empty	lysine-N-me	CBW17982.1	45 kDa	unknown	0	11	0	0	0	0	0	0	0	0	0	0
88	true	Empty	bifunctional	WP_000110806.1	89 kDa	unknown	0	0	0	0	0	13	0	0	0	0	0	0
89	true	Empty	phosphoetha	WP_001192092.1	66 kDa	unknown	0	0	0	0	0	0	0	0	0	13	0	0
90	true	Empty	MULTISPECIE	WP_001283356.1	70 kDa	unknown	0	0	0	0	0	0	0	0	0	0	0	11
91	true	Empty	outer memb	CBW19259.1	54 kDa	unknown	0	0	0	12	0	0	0	0	0	0	0	0
92	true	Empty	50S ribosom	CBW19492.1	14 kDa	unknown	0	0	0	0	0	0	0	10	0	0	0	0
93	true	Empty	CTP syntheta	WP_000210863.1	60 kDa	unknown	0	0	0	0	0	0	0	0	0	11	0	0
94	true	Empty	formate ace	CBW17006.1	85 kDa	unknown	0	0	0	0	0	10	0	0	0	0	0	2
95	true	Empty	ATP-depend	CBW19349.1 (+1)	70 kDa	unknown	0	0	0	0	0	11	0	0	0	0	0	0
96	true	Empty	hypothetical	CBW19752.1	43 kDa	unknown	0	10	0	0	0	0	0	0	0	0	0	0
97	true	Empty	cytoskeleton	WP_001090890.1	36 kDa	unknown	0	0	0	11	0	0	0	0	0	0	0	0
98	true	Empty	acetate kina	CBW18408.1	43 kDa	unknown	0	10	0	0	0	0	0	0	0	0	0	0
99	true	Empty	MULTISPECIE	WP_000036734.1	46 kDa	unknown	0	10	0	0	0	0	0	0	0	0	0	0
100	true	Empty	N-acetylglut	WP_000588964.1	49 kDa	unknown	0	0	0	8	0	0	0	0	0	0	0	0
101	true	Empty	1,4-alpha-gl	CBW19599.1	84 kDa	unknown	0	0	0	0	0	0	0	0	0	0	0	8
102	true	Empty	MULTISPECIE	WP_000462785.1	40 kDa	unknown	0	7	2	0	0	0	0	0	0	0	0	0
103	true	Empty	alcohol dehy	CBW17775.1	96 kDa	unknown	0	0	0	0	0	4	0	0	0	0	0	5
104	true	Empty	aconitate hy	CBW16261.1	94 kDa	unknown	0	0	0	0	0	5	0	0	0	0	0	2
105	true	Empty	D-alanyl-D-a	CBW16936.1	44 kDa	unknown	0	8	0	0	0	0	0	0	0	0	0	0
106	true	Empty	chemotaxis	CBW17950.1	73 kDa	unknown	0	0	0	0	0	0	0	0	0	0	0	8
107	true	Empty	UDP-glucose	CBW18153.1	44 kDa	unknown	0	6	0	0	0	0	0	0	0	0	0	0
108	true	Empty	topoisomera	CBW19254.1	70 kDa	unknown	0	0	0	0	0	0	0	0	0	0	0	8
109	true	Empty	possible pyri	CBW20163.1	52 kDa	unknown	0	0	0	7	0	0	0	0	0	0	0	0
110	true	Empty	phosphoenol	WP_000623114.1	63 kDa	unknown	0	0	0	0	0	0	0	0	0	7	0	0
111	true	Empty	non-heme fe	WP_000920611.1	19 kDa	unknown	0	0	0	0	0	0	0	8	0	0	0	0
112	true	Empty	SipA [Salmo	AAA86618.1 (+1)	74 kDa	unknown	0	0	0	0	0	5	0	0	0	0	0	0
113	true	Empty	transcription	CBW16908.1	18 kDa	unknown	0	0	0	0	0	0	0	6	1	0	0	0
114	true	Empty	Phenylalanyl	CBW17369.1	87 kDa	unknown	0	0	0	0	0	7	0	0	0	0	0	0
115	true	Empty	hypothetical	CBW18370.1	74 kDa	unknown	0	0	0	0	0	2	0	0	0	0	0	4
116	true	Empty	Porphyrin bi	CBW19980.1 (+1)	43 kDa	unknown	0	7	0	0	0	0	0	0	0	0	0	0
117	true	Empty	30s ribosom	CBW20412.1	9 kDa	unknown	0	0	0	0	0	0	0	7	0	0	0	0
118	true	Empty	acetylornithi	WP_000800210.1	42 kDa	unknown	0	7	0	0	0	0	0	0	0	0	0	0
119	true	Empty	tRNA/rRNA	WP_000997368.1	38 kDa	unknown	0	3	0	0	0	0	0	0	0	0	0	0
120	true	Empty	pyruvate deh	CBW16255.1	100 kDa	unknown	0	0	0	0	0	5	0	0	0	0	0	0
121	true	Empty	acriflavin res	CBW16570.1	42 kDa	unknown	0	6	0	0	0	0	0	0	0	0	0	0
122	true	Empty	hypothetical	CBW18402.1	45 kDa	unknown	0	6	0	0	0	0	0	0	0	0	0	0
123	true	Empty	predicted ba	CBW18695.1	40 kDa	unknown	0	6	0	0	0	0	0	0	0	0	0	0
124	true	Empty	Alanyl-tRNA	CBW18905.1	96 kDa	unknown	0	0	0	0	0	4	0	0	0	0	0	2
125	true	Empty	pathogenicit	CBW18962.1	62 kDa	unknown	0	0	0	0	0	0	0	0	0	0	0	5
126	true	Empty	2-oxoglutara	CBW16816.1	105 kDa	unknown	0	0	0	0	0	4	0	0	0	1	0	0
127	true	Empty	D-lactate de	CBW18241.1	65 kDa	unknown	0	0	0	0	0	0	0	0	0	5	0	0
128	true	Empty	probable N-a	CBW18515.1	32 kDa	unknown	0	0	0	0	0	0	0	4	0	0	0	0
129	true	Empty	phosphoglyc	CBW19144.1	41 kDa	unknown	0	4	0	0	0	0	0	0	0	0	0	0
130	true	Empty	cell division	CBW19630.1	54 kDa	unknown	0	0	0	0	0	5	0	0	0	0	0	0

131	true	Empty	DNA ligase (l	WP_000433266.1	73 kDa		unknown	0	0	0	0	0	0	0	0	0	0	0	5
132	true	Empty	DUF945 dom	WP_000753326.1	54 kDa		unknown	0	0	0	4	0	0	0	0	0	0	0	0
133	true	Empty	glycogen pho	WP_000993428.1	93 kDa		unknown	0	0	0	0	0	4	0	0	0	0	0	0
134	true	Empty	50S ribosom	CBW20176.1	15 kDa		unknown	0	0	0	0	0	0	0	5	0	0	0	0
135	true	Empty	Methionyl-tR	CBW18228.1	76 kDa		unknown	0	0	0	0	0	0	0	0	0	0	0	4
136	true	Empty	MULTISPECIE	WP_000081498.1	38 kDa	true	unknown	0	5	0	0	0	0	0	0	0	0	0	0
137	true	Empty	MULTISPECIE	WP_000271396.1	12 kDa		unknown	0	1	0	0	0	0	0	0	0	4	0	0
138	true	Empty	30S ribosom	CBW19498.1	10 kDa		unknown	0	0	0	0	0	0	0	3	0	0	0	0
139	true	Empty	assimilatory	WP_001290660.1	64 kDa		unknown	0	0	0	0	0	0	0	0	0	3	0	0
140	true	Empty	catalase HPII	CAB92320.1	84 kDa		unknown	0	0	0	0	0	4	0	0	0	0	0	0
141	true	Empty	NAD-linked r	CBW17591.1	63 kDa		unknown	0	0	0	0	0	0	0	0	0	4	0	0
142	true	Empty	HflK protein	CBW20383.1	46 kDa		unknown	0	4	0	0	0	0	0	0	0	0	0	0
143	true	Empty	MULTISPECIE	WP_000190499.1	50 kDa		unknown	0	4	0	0	0	0	0	0	0	0	0	0
144	true	Empty	bifunctional	WP_000200080.1	43 kDa		unknown	0	4	0	0	0	0	0	0	0	0	0	0
145	true	Empty	ubiquinone-c	WP_000815313.1	62 kDa		unknown	0	0	0	0	0	0	0	0	0	4	0	0
146	true	Empty	excinuclease	WP_001289464.1	68 kDa		unknown	0	0	0	0	0	0	0	0	0	0	0	4
147	true	Empty	NADH dehydr	CBW18394.1 (+1)	100 kDa		unknown	0	0	0	0	0	3	0	0	0	0	0	0
148	true	Empty	S-adenosylm	CBW19164.1	42 kDa		unknown	0	4	0	0	0	0	0	0	0	0	0	0
149	true	Empty	preprotein tr	CBW16239.1	102 kDa		unknown	0	0	0	0	0	3	0	0	0	0	0	0
150	true	Empty	flavodoxin-de	WP_000551825.1	41 kDa		unknown	0	3	0	0	0	0	0	0	0	0	0	0
151	true	Empty	MULTISPECIE	WP_001192954.1	65 kDa		unknown	0	0	0	0	0	0	0	0	0	0	0	3
152	true	Empty	MULTISPECIE	WP_000065257.1	13 kDa		unknown	0	0	0	0	0	0	0	3	0	0	0	0
153	true	Empty	cytoplasmic	CBW19547.1	45 kDa		unknown	0	0	0	0	0	0	0	0	0	0	0	3
154	true	Empty	MULTISPECIE	WP_000753958.1	49 kDa		unknown	0	3	0	0	0	0	0	0	0	0	0	0
155	true	Empty	Biosynthetic	CBW19161.1 (+1)	72 kDa		unknown	0	0	0	0	0	0	0	0	0	0	0	3
156	true	Empty	30S ribosom	CBW19488.1	14 kDa		unknown	0	0	0	0	0	0	0	2	0	0	0	0
157	true	Empty	type III restri	WP_000910371.1	73 kDa		unknown	0	0	0	0	0	0	0	0	0	0	0	3
158	true	Empty	redox-regula	WP_000505857.1	40 kDa		unknown	0	2	0	0	0	0	0	0	0	0	0	0
159	true	Empty	MULTISPECIE	WP_000877172.1	18 kDa		unknown	0	0	0	0	0	0	0	2	0	0	0	0

END OF FILE

Uncropped figures

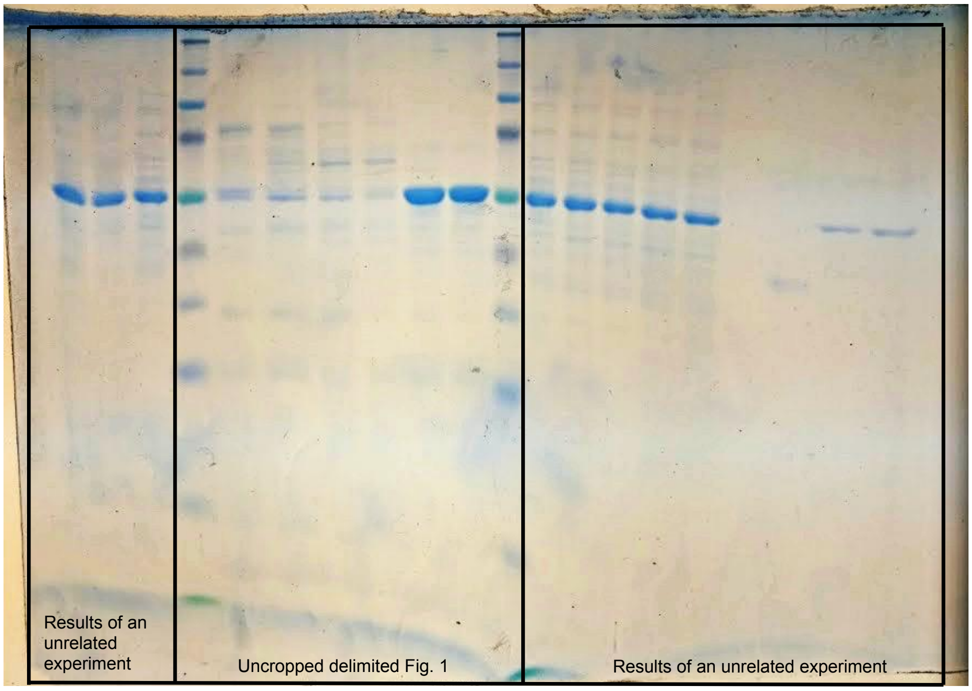

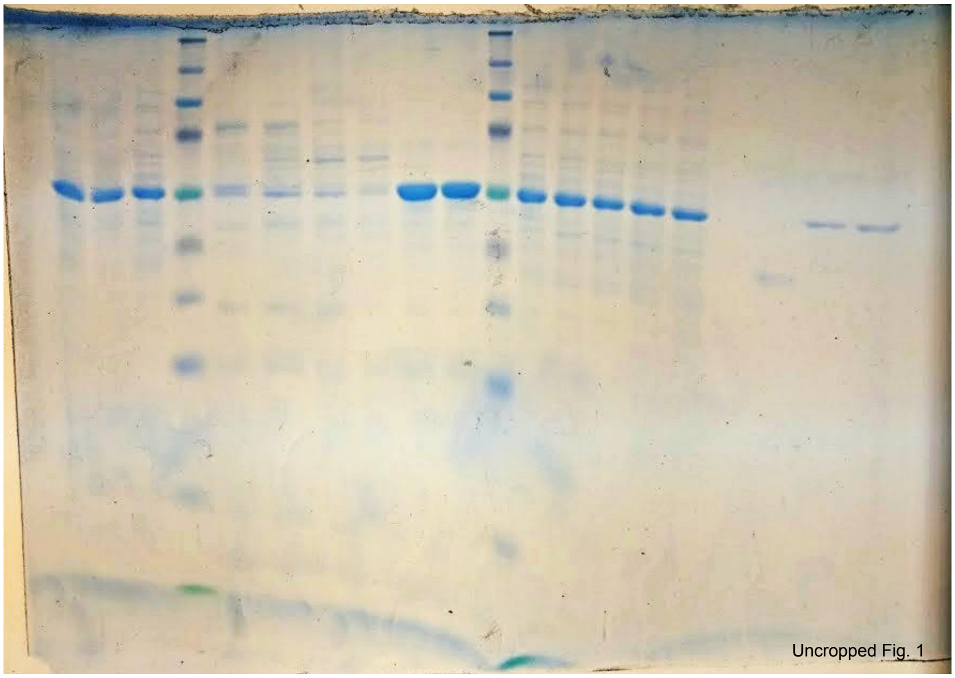

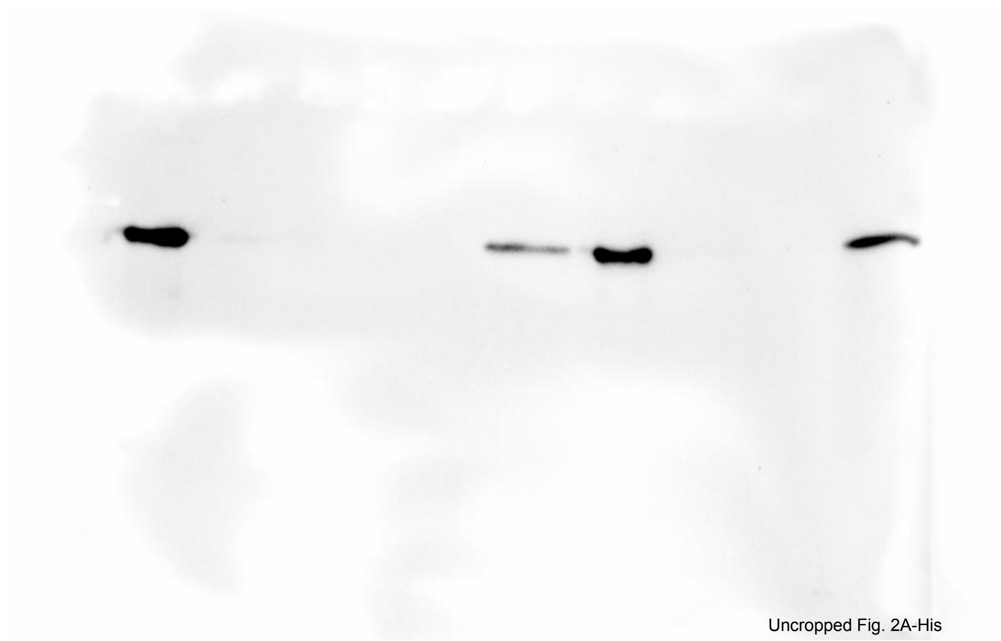
Uncropped Fig. 2A-His

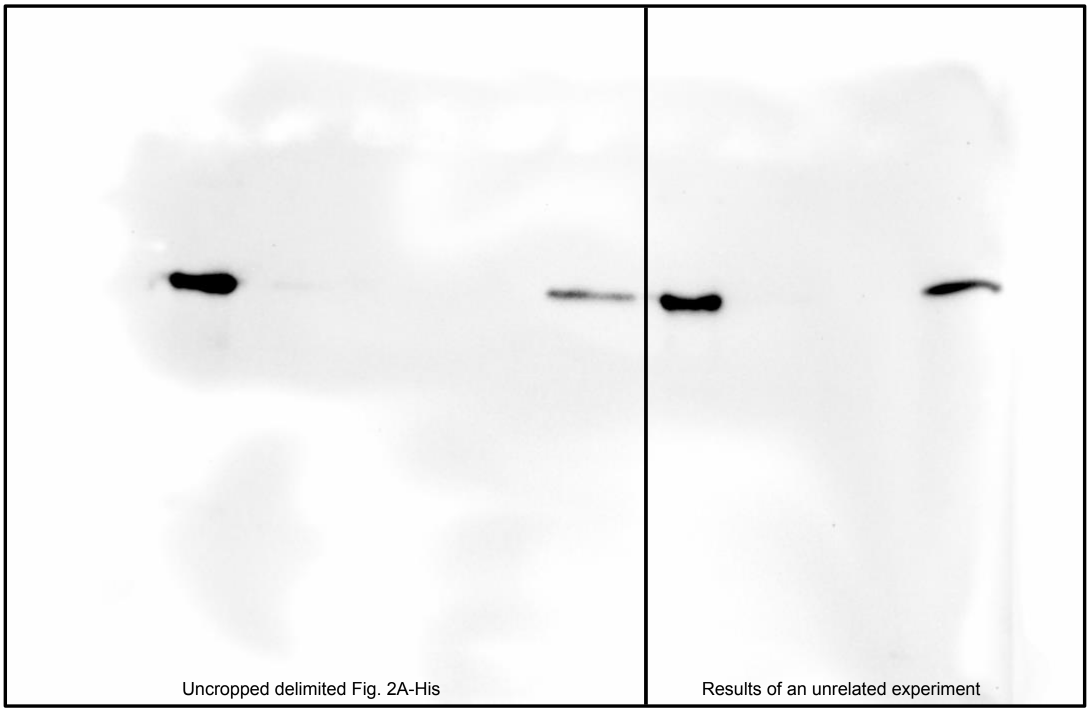
Uncropped delimited Fig. 2A-His
Results of an unrelated experiment

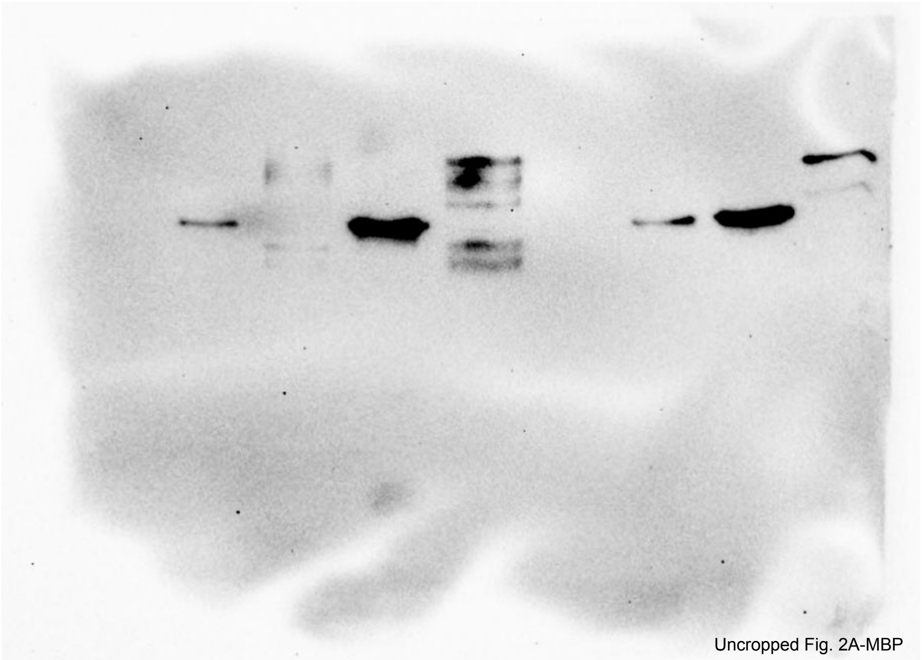
Uncropped Fig. 2A-MBP

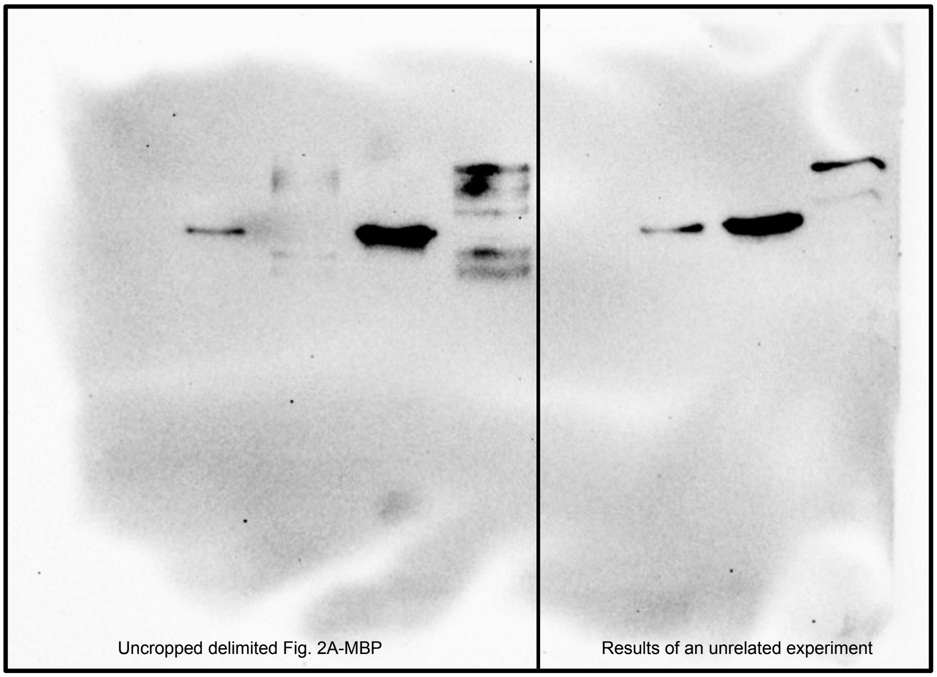
Uncropped delimited Fig. 2A-MBP
Results of an unrelated experiment

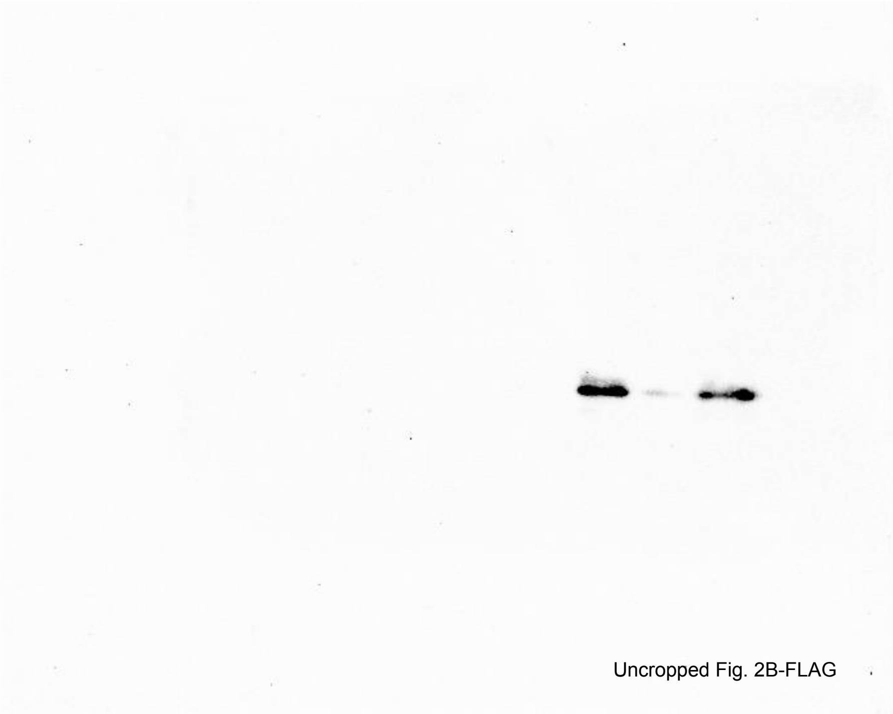

Uncropped Fig. 2B-FLAG

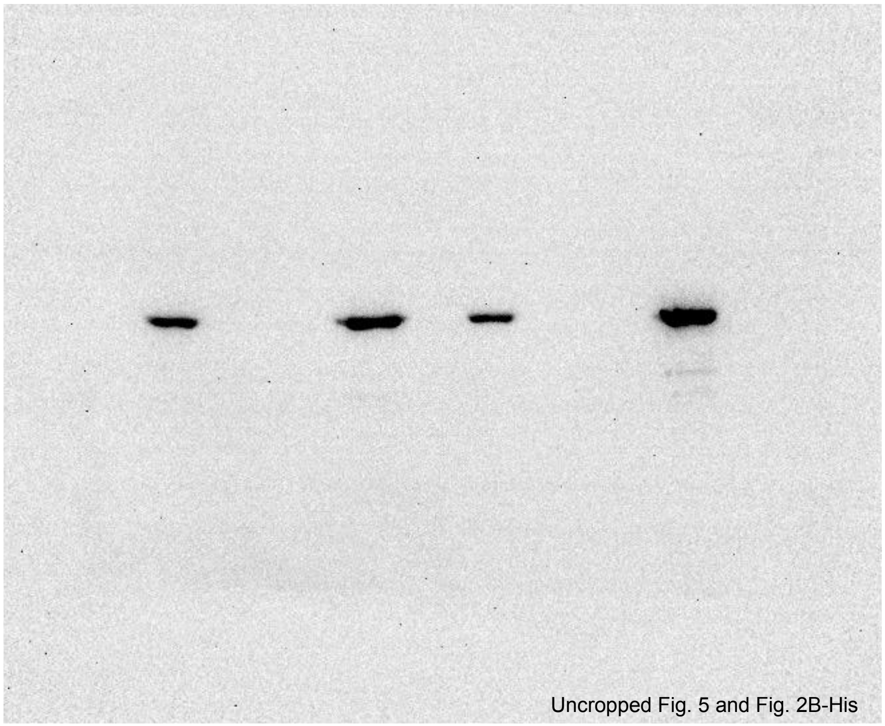

Uncropped Fig. 5 and Fig. 2B-His

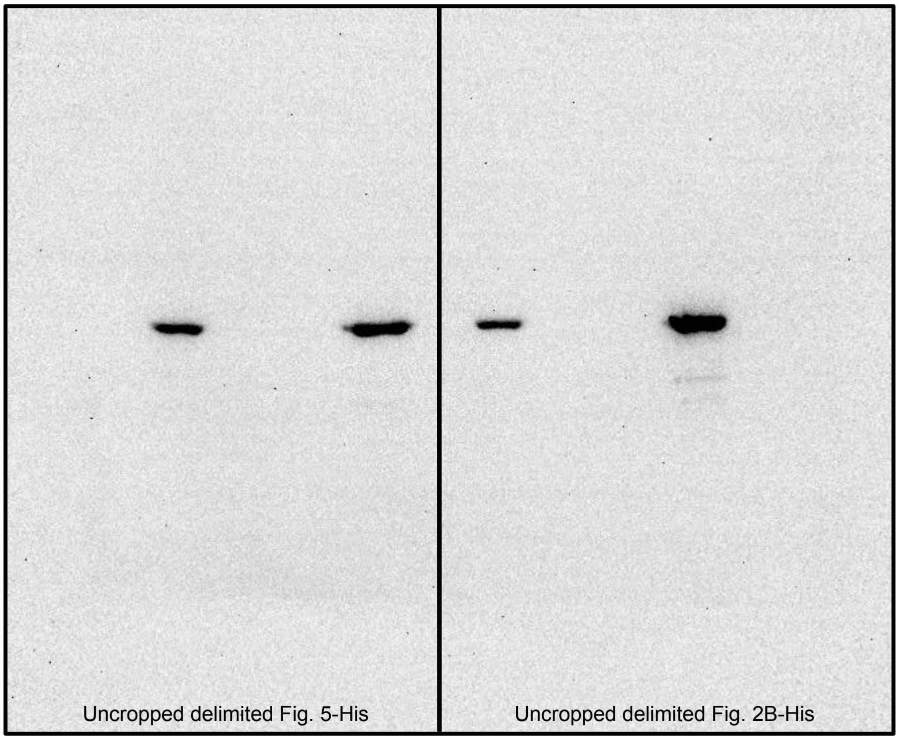

Uncropped delimited Fig. 5-His

Uncropped delimited Fig. 2B-His

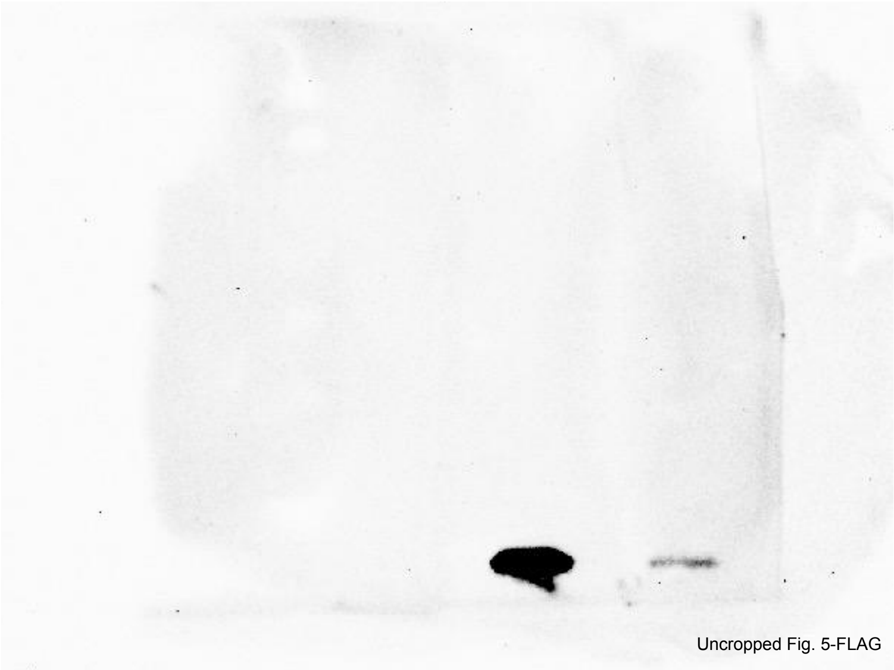

Uncropped Fig. 5-FLAG

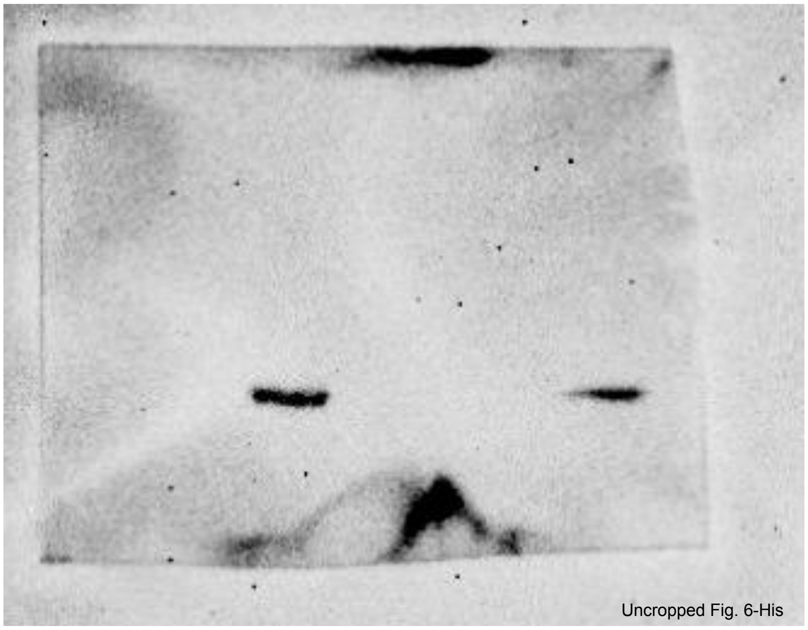
Uncropped Fig. 6-His

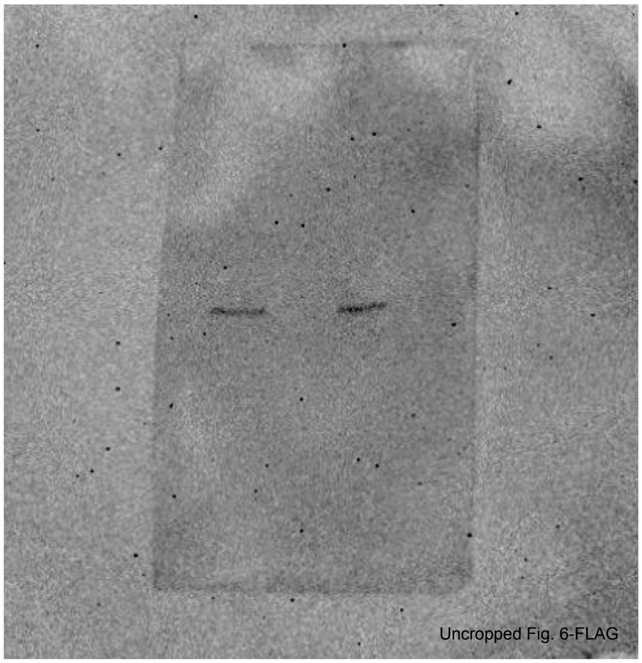
Uncropped Fig. 6-FLAG

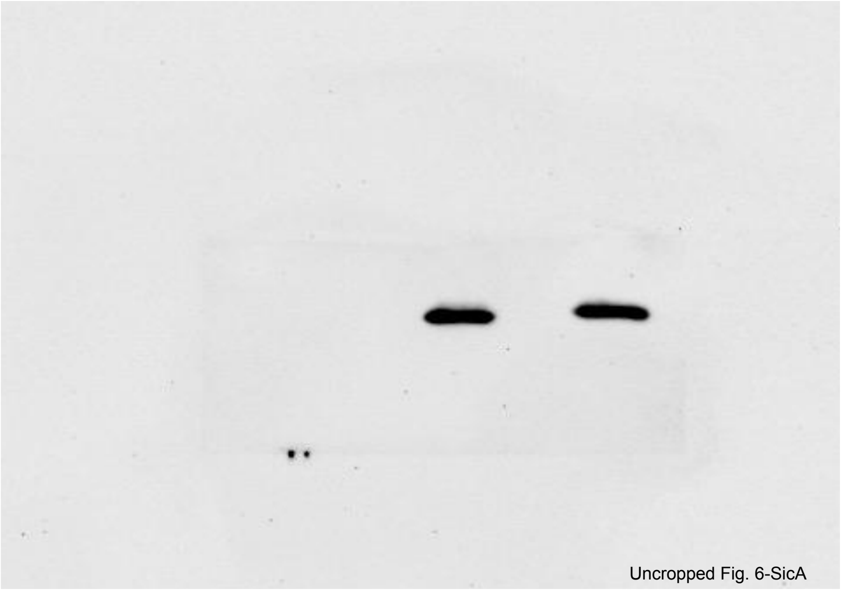
Uncropped Fig. 6-SicA

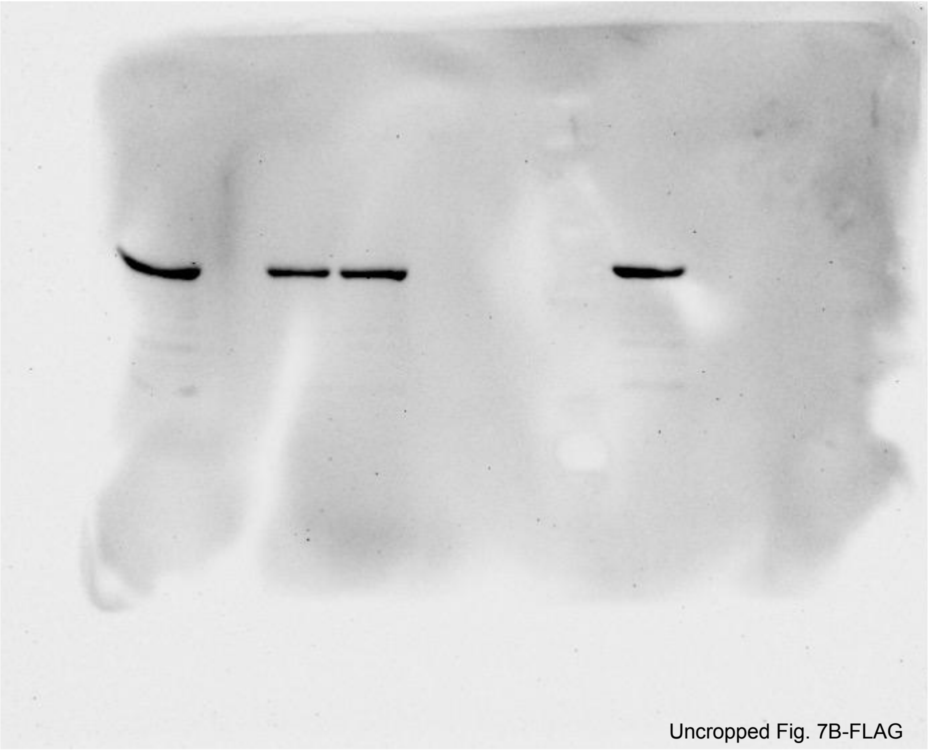
Uncropped Fig. 7B-FLAG

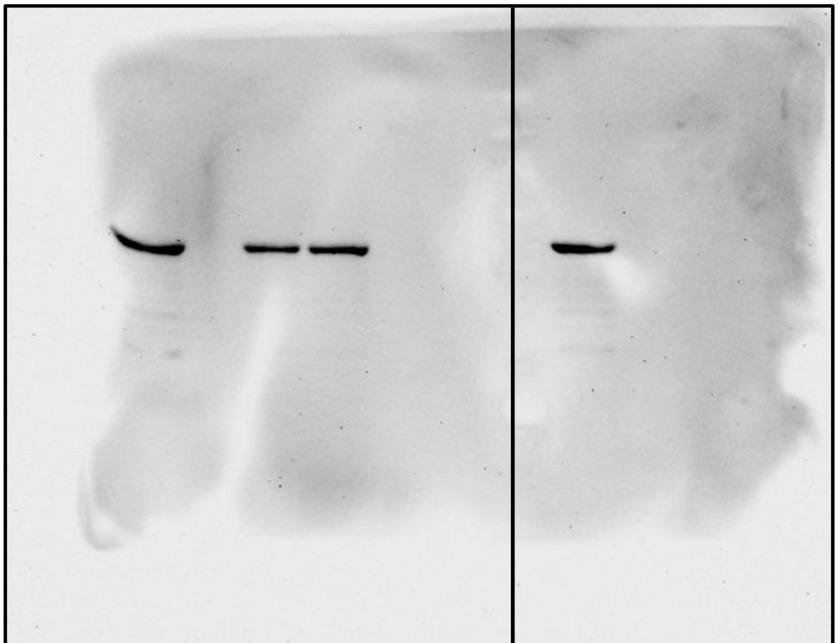

Uncropped delimited Fig. 7B-FLAG | Results of an unrelated experiment

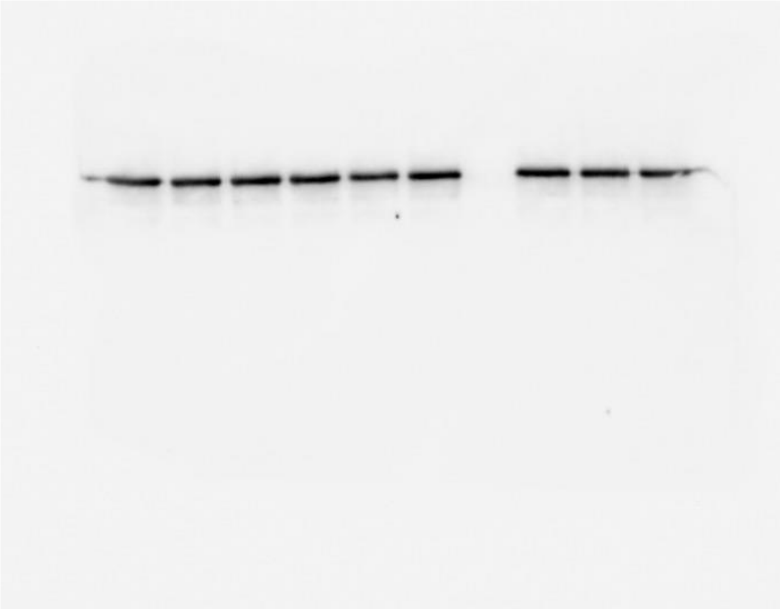

Uncropped Fig. 7B-GroEL

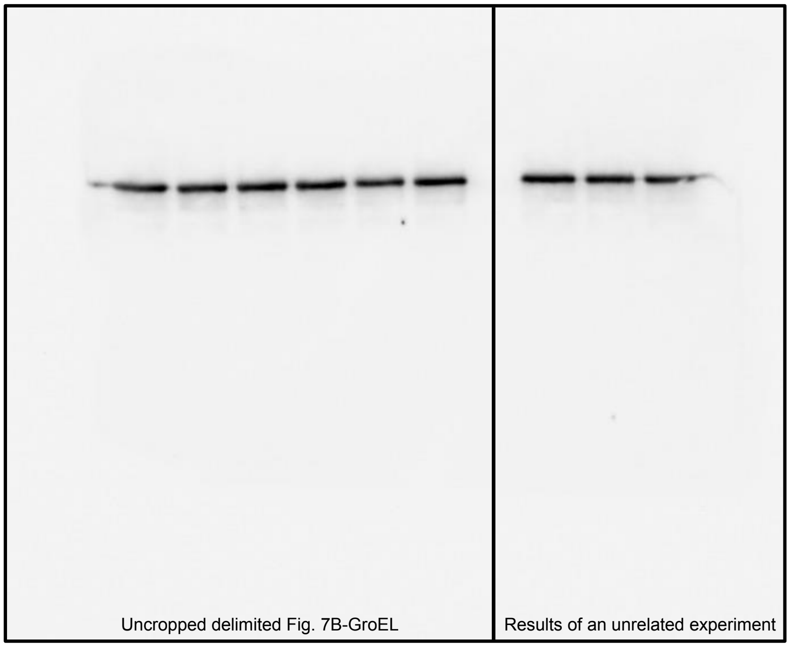

Uncropped delimited Fig. 7B-GroEL

Results of an unrelated experiment

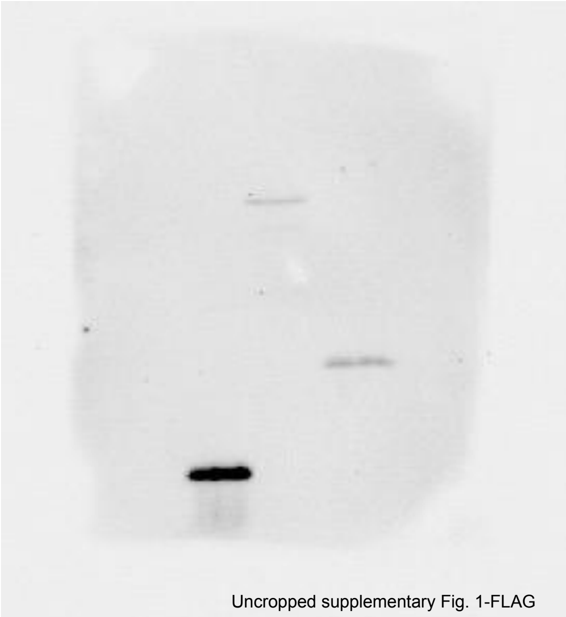

Uncropped supplementary Fig. 1-FLAG

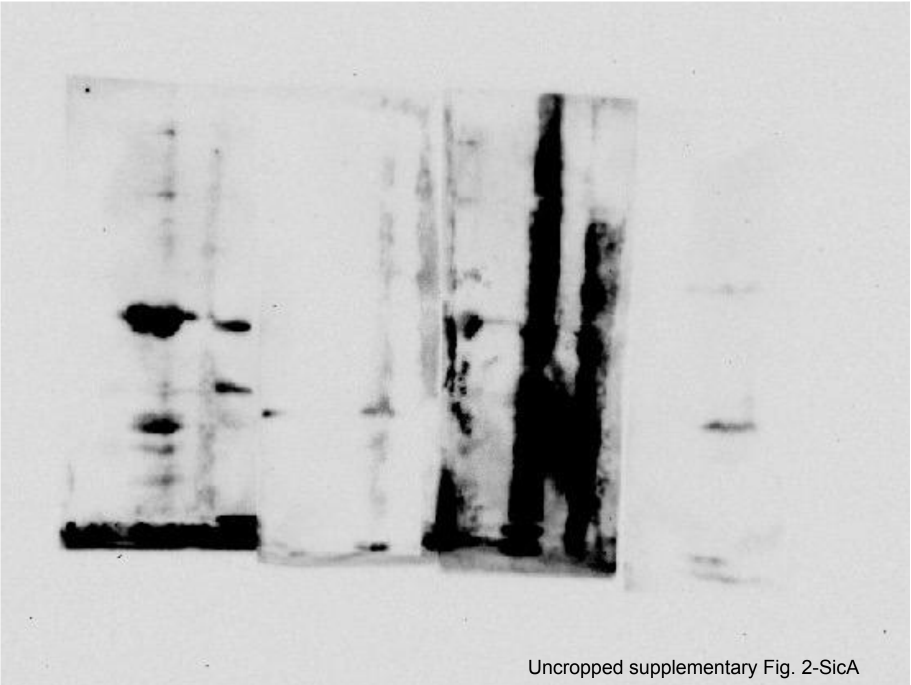
Uncropped supplementary Fig. 2-SicA

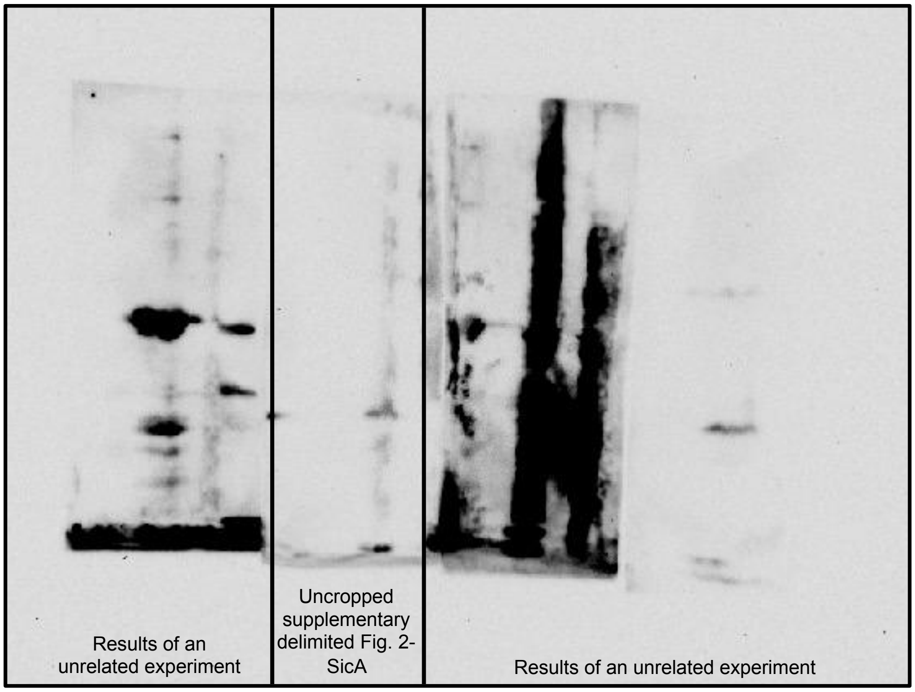
Results of an unrelated experiment
Uncropped supplementary delimited Fig. 2-SicA
Results of an unrelated experiment

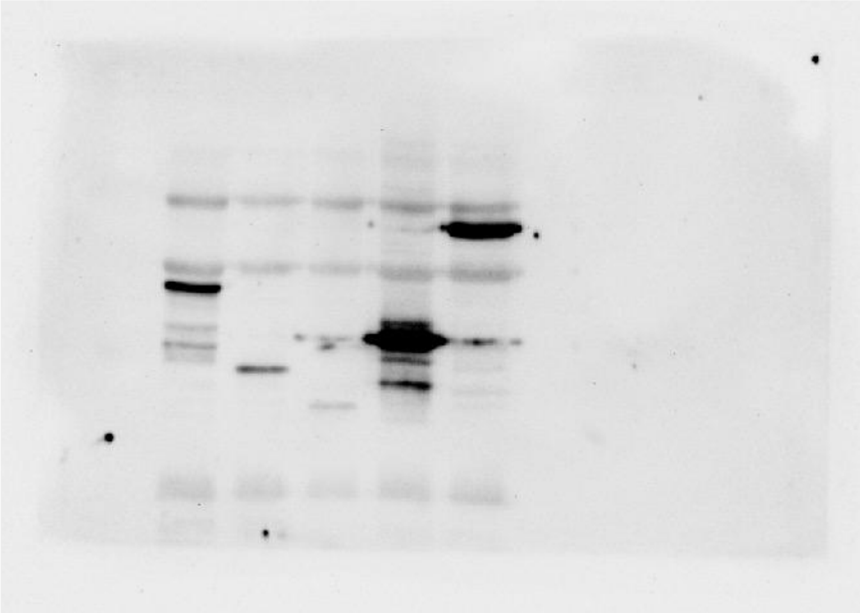

Uncropped supplementary Fig. 3-LexA
